# Characterization of the ExoU activation mechanism using EPR and integrative modeling

**DOI:** 10.1038/s41598-020-76023-3

**Published:** 2020-11-12

**Authors:** Maxx H. Tessmer, Samuel A. DeCero, Diego del Alamo, Molly O. Riegert, Jens Meiler, Dara W. Frank, Jimmy B. Feix

**Affiliations:** 1 Department of Chemistry, University of Washington, Seattle, WA USA; 2 Department of Microbiology and Immunology, Medical College of Wisconsin, Milwaukee, WI USA; 3 Department of Chemistry and Center for Structural Biology, Vanderbilt University, Nashville, TN USA; 4 Department of Molecular Physiology and Biophysics, Vanderbilt University, Nashville, TN USA; 5 Institute for Drug Discovery, Leipzig University Medical School, Leipzig SAC, Germany; 6 Department of Biophysics, Medical College of Wisconsin, Milwaukee, WI USA

**Keywords:** Biochemistry, Biophysics, Computational biology and bioinformatics

## Abstract

ExoU, a type III secreted phospholipase effector of *Pseudomonas aeruginosa*, serves as a prototype to model large, dynamic, membrane-associated proteins. ExoU is synergistically activated by interactions with membrane lipids and ubiquitin. To dissect the activation mechanism, structural homology was used to identify an unstructured loop of approximately 20 residues in the ExoU amino acid sequence. Mutational analyses indicate the importance of specific loop amino acid residues in mediating catalytic activity. Engineered disulfide cross-links show that loop movement is required for activation. Site directed spin labeling EPR and DEER (double electron–electron resonance) studies of *apo* and *holo* states demonstrate local conformational changes at specific sites within the loop and a conformational shift of the loop during activation. These data are consistent with the formation of a substrate-binding pocket providing access to the catalytic site. DEER distance distributions were used as constraints in RosettaDEER to construct ensemble models of the loop in both *apo* and *holo* states, significantly extending the range for modeling a conformationally dynamic loop.

## Introduction

Loop regions are often recognized as key regulatory elements of protein function, with their dynamic nature impacting characteristics such as enzyme activity^1,2^, substrate specificity^3,4^ and more^5^. Due to their intrinsic flexibility, the structural properties of protein loops are often difficult to study. Structural analyses for large proteins occur mainly through crystallography, and more recently cryo-electron microscopy, with the caveat that the conformation of loop regions often remain ambiguous. Several NMR based techniques can provide information on both structure and dynamics but are limited to relatively small proteins. In contrast, site directed spin labeling (SDSL) and electron paramagnetic resonance (EPR) spectroscopy are powerful tools that can be used to perform structural studies on dynamic protein regions^6^. SDSL EPR is not limited by protein size, and is a particularly valuable approach for the study of membrane proteins and complex macromolecular systems involving mutiple protein–protein interactions. Double electron–electron resonance (DEER) is an EPR technique for measuring the dipolar coupling of two spin centers^7^ to infer distance distributions between spin labels^8,9^. Obtaining a distance distribution is particularly advantageous for structural studies of flexible proteins because it can provide information on the dynamic range and relative populations of protein conformational states^10–12^. Experimental restraints obtained from DEER experiments have proven useful for integrative protein modeling. One of the most promising techniques couples DEER distances with the Rosetta molecular modeling suite^13,14^. This approach was used to develop high-resolution models of T4 lysozyme and αA-crystallin^15^. Another example is the use of RosettaDock and DEER distance restraints to identify the ubiquitin binding interface associated with the bacterial A2 phospholipase, ExoU^16^.

ExoU is a large (74 kDa) phospholipase A_2_^17–20^ expressed by a subset of strains of the opportunistic bacterial pathogen, *Pseudomonas aeruginosa*. In vivo synthesis of enzymatically active ExoU is correlated with acute lung injury^21^. ExoU expression is also essential for bacterial survival and replication, particularly within the first few hours of infection as assessed in animal models of *P. aeruginosa*-mediated disease^22^. Overall, multidrug resistance and ExoU production serve as significant biomarkers for severe disease and early mortality^23^. Understanding the structural events mediating the activation of ExoU's phospholipase activity is postulated to be a viable target for therapeutic intervention^24^.

Structural analysis of ExoU has been difficult. However, two nearly identical crystal structures were solved with ExoU in complex with its cognate chaperone, SpcU^25,26^. Approximately 25% of the coordinates are undetermined^25,26^ suggesting that ExoU is highly flexible, likely a necessary property for the protein to accommodate unfolding during translocation into the eukaryotic cytosol via the Type III secretion apparatus. Important functional regions of ExoU are absent from both structures, including a thirty-residue segment containing the catalytic aspartate (D344) and a thirteen-residue loop involved in PI(4,5)P_2_ binding and membrane localization^27–29^. While the interaction interfaces for ubiquitin^16^ and phosphatidylinositol-containing lipids^27^ are known, it is unclear how these interactions facilitate a shift of ExoU from an *apo* to a catalytically-active *holo* state, which are clearly distinguishable utilizing EPR approaches^28^.

In this study, SDSL-EPR and molecular modeling are used to gain a better understanding of the ExoU activation mechanism. The ExoU ubiquitin binding domain (UBD) was compared to the Rab5 binding domain of a homologous enzyme, VipD, a protein expressed by *Legionella pneumophila* that interferes with endosomal trafficking by depleting PI3P in a Rab5-dependent manner^30^. Rab5 is a member of the Ras-superfamily of small GTPase proteins. Based on structural comparisons, we postulated that an allosteric conformational change in an unresolved loop regulates activation of ExoU. The predicted conformational change was experimentally validated using continuous wave (CW) EPR to investigate local structural changes, DEER to show changes in conformational states, and engineered disulfide cross-linking to demonstrate the requirement for loop rearrangement. Using new molecular modeling techniques coupled with DEER restraints, we develop ensemble models of the flexible loop in both the *apo* and *holo* states.

## Results

### ExoU UBD shares structural homology to the VipD Rab5 binding domain

**Figure 1:**
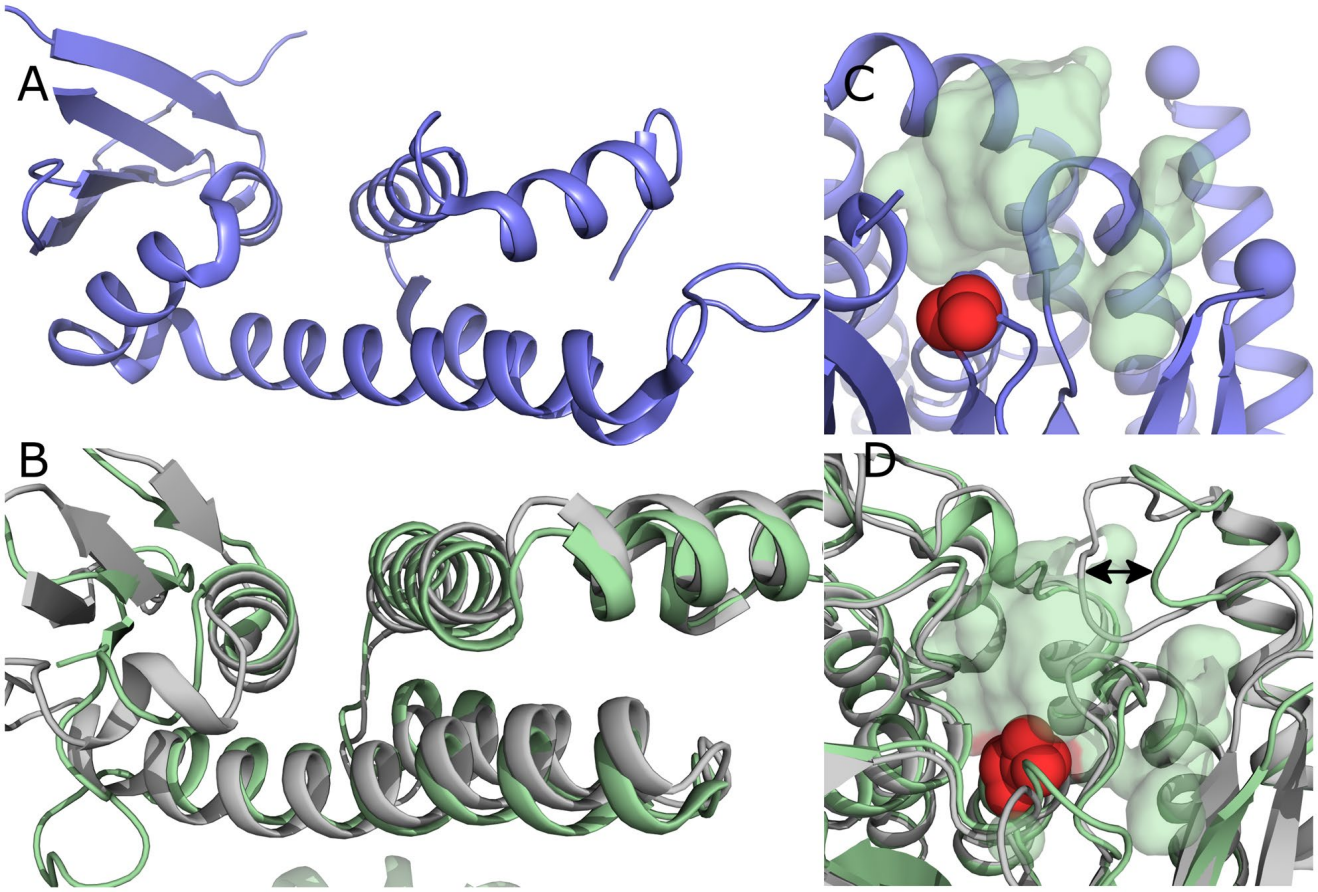
Homology between ExoU and VipD. (**A**) ExoU ubiquitin binding domain. (**B**) VipD Rab binding domain in the unbound (gray) and Rab5-bound (green) states. (**C**) ExoU catalytic domain. Red spheres represent the catalytic serine while the blue spheres represent the beginning and end of the unresolved loop homologous to the VipD lid. Transparent green surface is a representation of the VipD binding pocket superimposed on ExoU by structural alignment, illustrating the location of the hypothesized ExoU substrate binding pocket. (**D**) Allosteric conformational changes in the VipD catalytic domain. Red spheres represent catalytic serine. Transparent green surface represents the VipD binding pocket. Arrows indicate the VipD lid and conformational change in the Rab5-bound state (green) with respect to the unbound state (gray).

The phospholipase VipD, synthesized by *Legionella pneumophila*, is activated by Rab proteins^30^. Crystal structures of VipD in the *apo* state and in complex with Rab5 have previously been published^31,32^. Despite less than 20% sequence homology, ExoU and VipD share a structurally conserved fold. The α/β hydrolase fold of the ExoU and VipD catalytic domains is conserved amongst several lipases^33^, but the cofactor binding domains are not. Comparison of the VipD Rab binding domain (RBD) with the ExoU UBD reveals modest structural homology (Fig. 1A,B) with a root mean squared deviation (RMSD) of 3.9 Å over 104 residues. By analyzing conformational differences between the Rab5 bound and unbound states, Lucas et al*.*^32^ proposed an activation mechanism for VipD. Briefly, the binding of Rab5 provokes a conformational change in the catalytic domain of VipD, allosterically displacing a loop that covers the active site and forming a pocket that could potentially accommodate the acyl chain of a lipid substrate (Fig. 1D). This mechanism of activation was proposed based on crystal structures, but no further experimental validation was performed^32^. Given the homology of the ExoU UBD to the VipD RBD, we postulated that ExoU may share a similar activation mechanism. Notably, the analogous loop (residues 427–446) of ExoU lacks coordinates in either ExoU crystal structure (Fig. 1C), suggesting the loop is dynamically disordered in the *apo* state^25,26^. This flexibility may be a key structural feature that allows ExoU to transition from an inactive state to an active state, and to interact with a variety of phospholipid substrates. Lucas et al*.*^32^ refer to the regulatory loop of VipD as a “lid”; however, in other phospholipases such as cPLA2, this term often refers to a different region regulating interfacial activation^34^. To avoid confusion, we will refer to the proposed regulatory loop of ExoU by its amino acid position as loop_427–446_.

### ***Displacement of loop***_***427–446***_*** is required for phospholipase activity***

**Figure 2:**
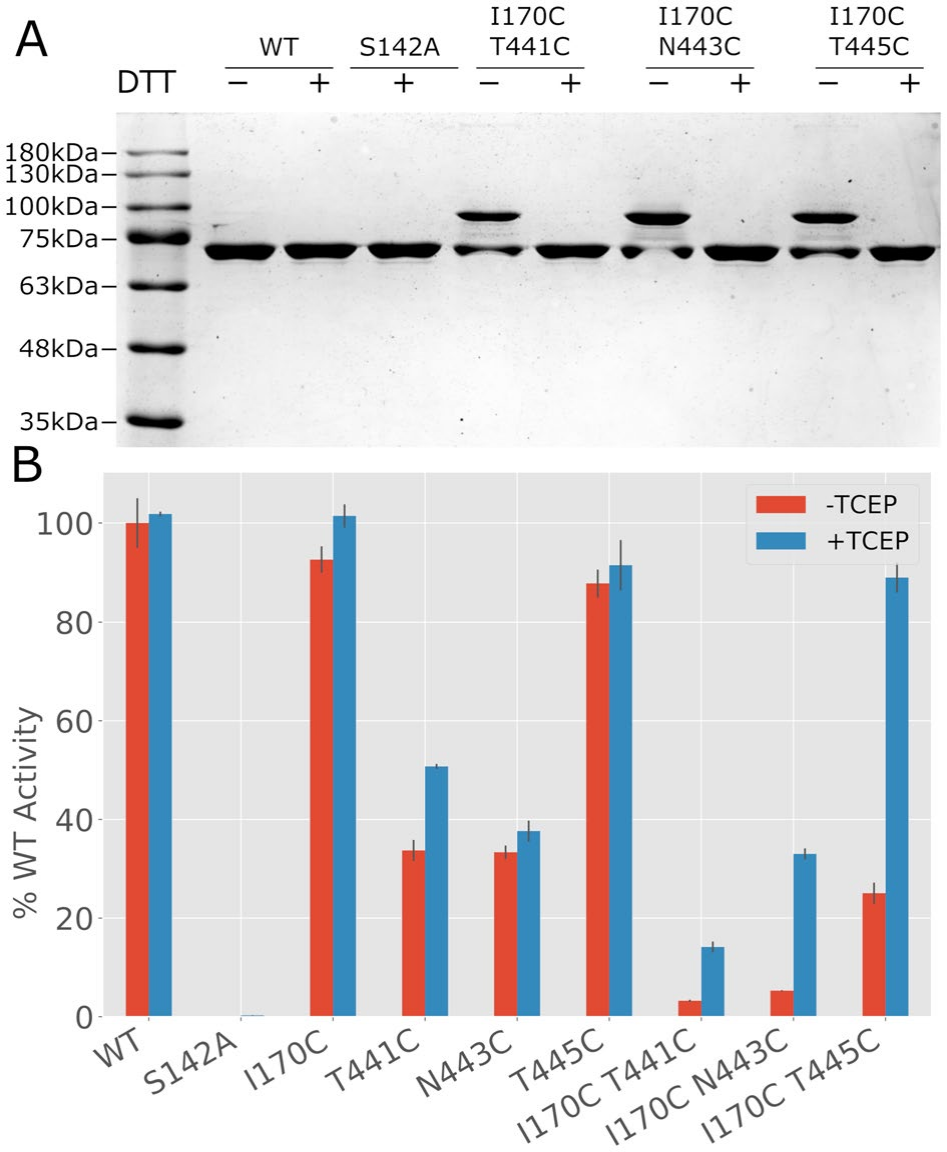
EMSA of ExoU double cysteine mutants (**A**) EMSA of ExoU disulfide cross links in the presence and absence of DTT. Each lane contains approximately 1 µg of protein loaded onto a sodium dodecyl sulfate (SDS) polyacrylamide (10%) gel, stained with Coomassie blue and imaged using a Biorad ChemiDoc set on autocapture. (**B**) Normalized activities of ExoU disulfide cross linked variants in the presence or absence of TCEP. WT and S142A serve as positive and negative controls respectively.

We postulate loop_427–446_ performs two functions that together facilitate enzymatic activity when ubiquitin and substrate interact with ExoU. Structural analogy to VipD suggests that loop_427–446_ may act as a gate to occlude the ExoU active site when closed, and part of the substrate binding pocket when open. To test this hypothesis, we designed three constructs, each containing cysteine substitutions at a loop residue (T441C, N443C or T445C) and at a site on the opposite side of the proposed substrate binding pocket (I170C). If our hypothesis is correct, these cysteine residues should be in close enough proximity to form disulfide bonds. The formation of a disulfide bridge is postulated to make ExoU phospholipase activity dependent on a reduction step to open the proposed binding pocket. Each ExoU derivative was purified and an electrophoretic mobility shift assay (EMSA) in the presence and absence of dithiothreitol (DTT) was performed. All three constructs show a significant electrophoretic mobility shift in the absence of DTT with respect to wild type ExoU (Fig. 2A). These higher molecular weight species were eliminated in the presence of DTT, suggesting that they resulted from the designed disulfide bond. We also performed an EMSA on the single-cysteine controls (Supplementary Fig. S1). A small population of mobility shifted bands is present near the top of the gel for each of the single cysteine mutants, likely due to the formation of intermolecular disulfide bonds. These bands were eliminated in the presence of DTT.

After validation by EMSA, constructs were tested for enzyme activity (Fig. 2B) with and without preincubation in 2 mM tris (2-carboxyethyl) phosphine (TCEP). Notably, the single-cysteine controls all have small increases in activity upon the addition of TCEP, likely due to the reduction of intermolecular disulfides as described above. For each of the double-cysteine constructs, substantially larger increases in activity were observed upon reduction with TCEP. For both I170C-N443C and I170C-T445C activity following TCEP reduction was similar to the N443C and T445C single mutants, respectively, suggesting efficient reduction of the disulfide bond. In contrast, the activity of I170C-T441C following reduction was significantly less than that for reduced T441C, suggesting that either this disulfide bond is less accessible to TCEP or that the combined effect of the I170C-T441C mutations is more detrimental than either mutation alone. Overall, these data show that activation of ExoU requires a conformational change in loop_427–446_ that displaces it relative to the adjacent helix containing I170. These results are consistent with the hypothesis that movement of loop_427–446_ opens a substrate binding pocket facilitating phospholipase activity.

### ***The composition of loop***_***427–446***_*** is important for phospholipase activity***

To test the hypothesis that loop_427–446_ plays a structural role in the formation of a postulated binding pocket, five mutations were designed to replace residues deemed likely to be important in defining the loop’s structure and/or function. Three of these mutations (T441D, F444D, M446D) were designed to reduce the hydrophobicity of the hypothesized pocket, thereby disrupting potential hydrophobic interaction with alkyl chains of a phospholipid substrate. Additionally, we sought to disrupt the overall structure of the loop by introducing a bulky arginine in place of conserved glycine (G440R) or introducing a proline to disrupt the backbone structure (N443P). Recombinant proteins were made for each construct and tested for activity using the fluorogenic phospholipid analog, PED6. Catalytic activity was reduced for each point mutant as compared to WT ExoU, as shown by reductions in *V*_*max*_ and increased *K*_*m*_ values (Table 1), supporting the hypothesis that loop_427–446_ is important for maintaining enzymatic activity.

**Table 1:** Kinetic constants and fit parameters for ExoU loop_427–446_ point mutation variants.

Variant	*K*_*m*_ (µM PED6)		*V*_*max*_ (nmol PED6 min^−1^)	
WT ExoU	12.2	± 5.1	0.0694	± 0.0078
G440R	66.5	± 13.0	0.0243	± 0.0020
T441D	28.0	± 6.9	0.0047	± 0.0004
N443P	28.4	± 4.9	0.0313	± 0.0018
F444D	46.2	± 8.3	0.0166	± 0.0011
M446D	37.3	± 4.8	0.0208	± 0.0009

### ***ExoU loop***_***427–446***_*** undergoes a conformational change upon activation***

**Figure 3:**
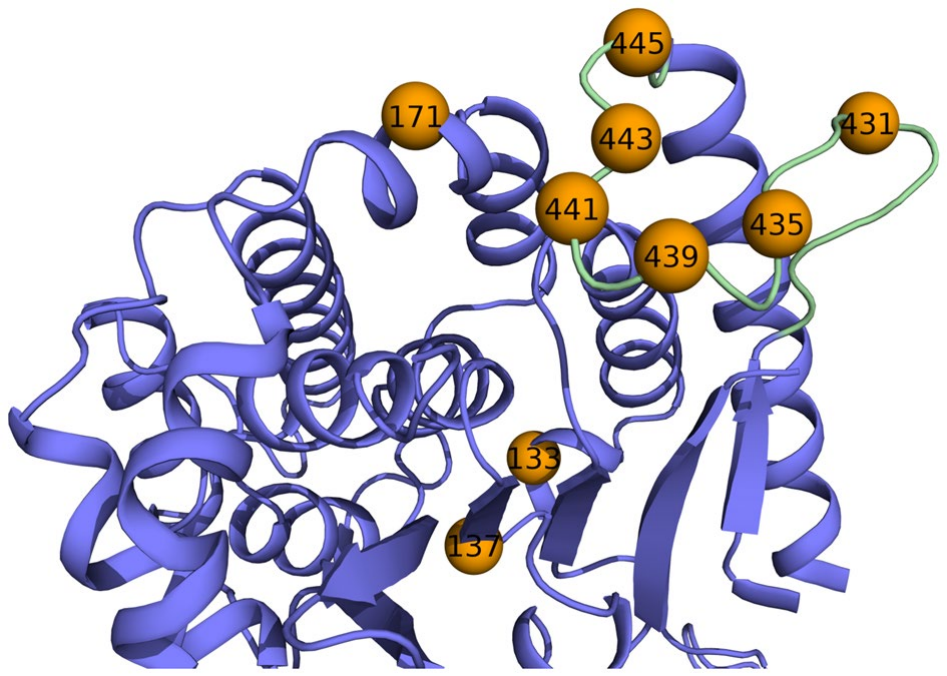
Labeling sites for SDSL and DEER. Model depicting the positions of cysteine substitutions used for site-directed spin labeling. Residues 133 and 137 are observed in the ExoU-SpcU crystal structures^25,26^. Loop_427–446_ was modeled into PDB 3TU3^26^ using Rosetta.

To investigate putative conformational changes in loop_427–446_ upon activation, we constructed seven double-cysteine variants of ExoU for DEER analysis via SDSL-EPR (Fig. 3). One cysteine was introduced at a well-studied reference site, S137C^35^, and paired with six individual cysteine substitutions within loop_427–446._ Recombinant ExoU variants were purified and the introduced cysteine residues were spin-labeled to generate the R1 nitroxide side chain (Supplementary Fig. S2A^36^). To ensure that observations were not exclusive to the S137R1 reference site, a double cysteine variant with the reference site at D133R1 was also constructed. All of the single mutation variants with cysteine residues introduced into loop_427–446_ retained catalytic activity, both before and after spin labeling (Supplementary Fig. S2C). Notably, activities of 133C and 137C and their spin-labeled (R1) variants were comparable to that of wild type ExoU.

**Figure 4:**
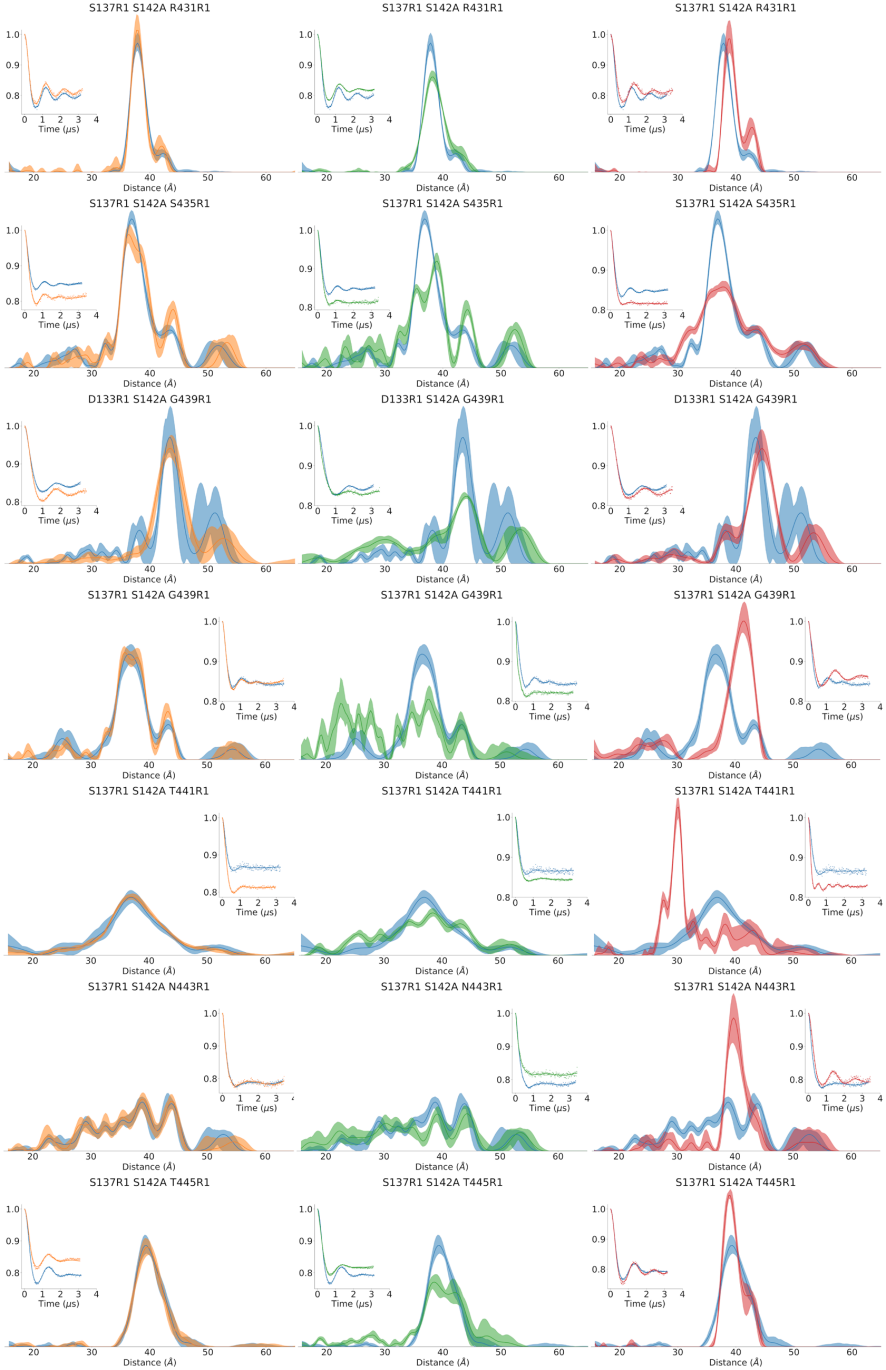
Intermolecular DEER of the ExoU loop_427–446_. DEER distance distributions were determined for double spin labeled ExoU in the apo state (blue), in the presence of diubiquitin alone (orange), in the presence of nanodiscs alone (lipo state, green) or in the presence of both (holo state, red). Each trace compares the apo state to each of the other conditions. Insets are the background corrected (3D) time domain data of the DEER experiment. Error bands were calculated using the DeerAnalysis 2018 default validation protocol.

DEER distances were collected for ExoU alone (*apo*), or in the presence of diubiquitin (diUb), nanodiscs (*lipo*) or both (*holo*). Linear diUb was used in all experiments to saturate ExoU and drive the equilibrium towards an active state, since this ubiquitin isoform is known to activate ExoU with high efficiency^37^. To prevent degradation of substrate lipid bilayers (nanodiscs) all constructs were made in the catalytically inactive S142A ExoU background. Loop_427–446_ DEER data are summarized in Fig. 4 and Table 2. In the *apo* state, loop_427–446_ shows relatively narrow distance distributions (P_max_ > 0.1) near the N terminal half of the loop (e.g. R431R1, S435R1) and broader distributions (P_max_ < 0.1) near the C-terminal half (e.g. T441R1 and N443R1). The addition of diUb alone had little to no effect on the distance distributions (Fig. 4, column 1), consistent with our previous observation of a required synergistic interaction with substrate and cofactor for the production of ExoU conformational changes^28^. Upon the addition of nanodiscs, all distance distributions broadened, suggesting destabilization of loop_427–446_ upon membrane association (Fig. 4, column 2). With the addition of both nanodiscs and diubiquitin (*holo* state) all distance distributions narrowed except 137R1-435R1 (Fig. 4, column 3), and several sites (e.g. 439R1, 441R1, 443R1) exhibited distance distributions that were significantly different than those observed in the *apo* state. Taken together these data show that at least some regions of loop_427–446_ occupy a relatively restricted conformational space even in the *apo* state, and that formation of the *holo* state stabilizes a new loop conformation. These conformational changes are particularly evident in the time domain data of the DEER experiment (Fig. 4, insets). Notably, the C-terminal half of loop_427–446_ appears to become significantly more rigid in the *holo* versus the *apo* state, as indicated by well-defined oscillations in the time domain data.

**Table 2:** Summary of DEER data.

	Apo		diUb		Lipo		Holo	
	Peak^a^	P_max_^b^	Peak	P_max_	Peak	P_max_	Peak	P_max_
S137R1 S142A R431R1	37.9	0.27	37.7	0.29	38.3	0.20	39.0	0.28
S137R1 S142A S435R1	36.8	0.14	36.4	0.12	39.0	0.10	37.7	0.07
D133R1 S142A G439R1	43.5	0.14	43.5	0.12	44.0	0.07	44.5	0.12
S137R1 S142A G439R1	36.5	0.12	35.8	0.12	37.8	0.07	41.6	0.16
S137R1 S142A T441R1	36.9	0.08	36.9	0.08	38.5	0.06	30.3	0.21
S137R1 S142A N443R1	38.8	0.07	38.8	0.07	44.3	0.05	39.8	0.19
S137R1 S142A T445R1	39.4	0.17	39.7	0.16	38.5	0.11	38.8	0.25

^a^Maximum probability distance (Å).

^b^Maximum probability of the normalized distribution (Å^−1^). Large values indicate sharp peaks while low values indicate broad peaks.

CW EPR spectra for single-cysteine loop variants in an S142A background were also collected and analyzed (Supplementary Fig. S2B). These data are consistent with the observed DEER data, as they show small conformational changes in the presence of nanodisc bilayers alone and much larger changes upon the addition of both nanodiscs and diUb. Notably, rotational mobility of the spin label is substantially reduced in the C-terminal region of the loop (at S439R1, T441R1, N443R1 and T445R1), consistent with decreased conformational flexibility. CW EPR spectra of S137R1 show relatively little deviation, suggesting that the reference site does not undergo significant conformational changes upon adoption of the *holo* state, confirming that the observed differences in the DEER data are a result of conformational changes in loop_427–446_. None of the sites showed any change upon incubation with the MSP1D1 scaffold protein in solution (Supplementary Fig. S3). In addition, we verified that the conformational changes observed in the lipo and holo states with liposomes were identical to those observed with nanodiscs (Supplementary Fig. S4). These data indicate that ExoU interacts with the lipid bilayer portion of the nanodisc and not with the scaffold protein.

### ExoU loop_427–446_ does not insert into the lipid bilayer

Changes observed in DEER distance distributions upon the addition of nanodiscs alone suggest that the labeled residues could potentially be interacting directly with the membrane. Interaction of loop_427–446_ residues with the membrane would be consistent with the hypothesized orientation of ExoU on the membrane surface^28^. To test this hypothesis, we used power saturation SDSL-EPR to screen four loop_427–446_ sites for insertion into the lipid bilayer (Supplementary Fig. S5). Although S435R1, G439R1 and N443R1 exhibited an increase in the EPR depth parameter in the *lipo* and *holo* states, Φ values remained negative suggesting that none of these sites insert into the membrane^38^. Notably, none of these sites exhibit an increase in the oxygen accessibility parameter, Π(O_2_). Instead, changes in Φ values are driven by decreased NiEDDA accessibility at all sites except R431R1 (Supplementary Fig. S5). Taken together these data indicate that S435R1, G439R1 and N443R1 experience a reduction in solvent accessibility in the *lipo* and *holo* states but are not inserting into the membrane.

### ExoU helix_168–176_ undergoes a conformational change in coordination with loop_427–446_

Given that cysteine residues in loop_427–446_ spontaneously form disulfide bonds with I170C, we postulate that ExoU helix_168–176_ forms the opposite side of the proposed substrate binding pocket. In VipD the corresponding region forms a channel leading to the catalytic site. To test if ExoU helix_168–176_ experiences a conformational change upon membrane binding and formation of the *holo* state, DEER experiments were performed utilizing S137R1-S171R1 ExoU in an S142A background under the four experimental conditions previously described (Supplementary Fig. S6). The side chain of S171 is located on a solvent exposed surface of the helix such that introduction of the R1 side chain should not impact local packing. In the *apo* state we observed a mode distance of 36.3 Å, similar to the simulated mode distance of 35.7 Å (Supplementary Fig. S7). Addition of diUb alone resulted in no change, as observed for loop_427–446_ sites. Addition of nanodiscs resulted in a broadening of the distribution and the appearance of a lower distance peak at about 24 Å. The distance distribution of S137R1–S171R1 in the *holo* state was substantially different than that seen in other states, with two narrow populations at 27 Å and 33 Å (Supplementary Fig. S6). Given the short distance between the two peaks, they may represent a single backbone conformation with two predominant side chain conformations of the R1 spin label. Cumulatively, these data indicate that S171R1 and the accompanying region undergo conformational changes upon membrane association and formation of the *holo* state. These changes, in addition to those observed for loop_427–446_, may aid the opening of the putative substrate binding pocket. Accordingly, we expect that changes in helix_168–176_ to also be involved in the gating mechanism of the ExoU active site. Alternatively, the changes in the S137R1–S171R1 distance distribution observed in the presence of lipids alone could be due to steric interaction with the membrane surface, altering the rotamer distribution of the spin label. This explanation is consistent with the decrease in spin label solvent exposure observed in power saturation experiments. However, interaction with the membrane surface alone does not account for the distinct, narrow distance distributions observed in the holo state.

### Modeling the active conformation of ExoU Loop_427–446_

To gain a better understanding of the active structure of ExoU we modeled the *apo* and *holo* conformations of loop_427–446_ using Rosetta. The workflow for protein and ensemble modeling is summarized in Supplementary Fig. S8. Notably, loop_427–446_ is nearly twice as large as the typical upper limit of Rosetta de novo loop modeling^39^. A homologous enzyme from the organism *Pseudomonas fluorescens* (*pf*ExoU) was recently crystalized (PDB: 4QMK)^29^, and while the conformation of the analogous *pf*ExoU loop_375–395_ is only partly determined, some of the N-terminal residues have defined coordinates. This is consistent with our DEER results indicating that the N-terminal half of ExoU loop_427–446_ is less flexible. Additionally, since the N-terminal residues of ExoU loop_427–446_ show little conformational change upon transition to the *holo* state (Fig. 4, Table 2), we used *pf*ExoU loop_375–394_ as a template for modeling the *holo* state as well. Importantly, *pf*ExoU is 44.7% identical to ExoU over 657 amino acids, and ExoU loop_427–446_ is 61.9% identical to *pf*ExoU loop_375–395_. In addition to serving as a template, the conserved nature of this loop suggests an important function, consistent with our experimental observations. To further enhance our modeling we utilized the RosettaDEER module, allowing full use of the DEER distance distributions^40^.

To generate a pool of models of ExoU structures with loop_427–446_, we used RosettaCM^41^, a homology modeling module of the Rosetta protein modeling software suite, with the 3TU3 and 4QMK PDB structures as templates (“Materials and methods”). There are several caveats to the modeling of ExoU loop_427–446_ that should be noted. First, we chose to use the *apo* state restraints for helix_168–176_ and downstream residues 180–209 in modeling the holo state despite the likelihood that these regions may undergo significant conformational changes. This was needed because segments containing these regions are too large to model de novo without additional restraints, and their absence would likely cause significant scoring artifacts perturbing the overall structure of the models. For this reason, the S137R1–S171R1 distance restraint was omitted from ensemble modeling of the *holo* state. Another caveat is the absence of a membrane model in the *holo* state. This could be significant due to likely orthosteric effects on ExoU near the catalytic domain as well as potential steric effects on the spin labels that could bias the distance distributions. Nonetheless, given the experimental evidence that loop_427–446_ fails to insert into the bilayer we posit that it should be possible to model its conformation with DEER distributions as primary constraints. Finally, it is important to consider the fact that the introduction of the spin labels themselves may introduce structural perturbations to ExoU and that each experimental distribution may correspond to a slightly different backbone structure. However, we anticipate that the simultaneous fitting of all the distance distributions will dilute the unique contributions of any one construct and drive the modeling towards a structural average.

Three ensemble models each for the *apo* and *holo* states were prepared as described in the materials and methods section. Ensembles are represented as a set of protein structures with weights corresponding to the relative populations of each structure within the ensemble. Simulated ensemble distance distributions are linear combinations of the weighted distance distributions of each member of the ensemble:

$$ P_{ens} \left( r \right) = \mathop \sum \limits_{i} w_{i} P_{i} \left( r \right). $$

**Figure 5:**
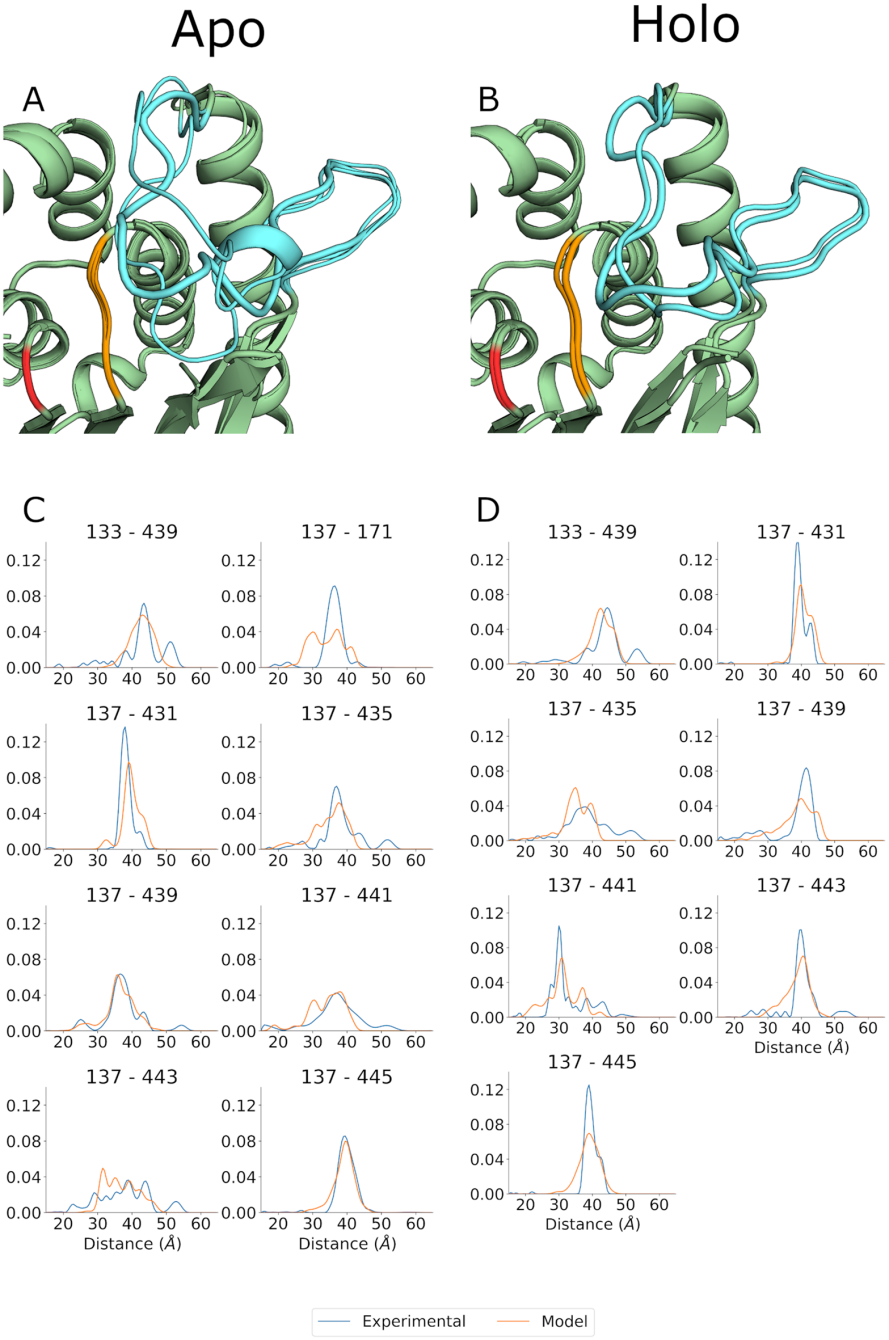
ExoU loop_427–446_ ensemble modeling. Best fitting ensembles are shown for the apo (left) and holo (right) state of ExoU loop_427–446_. (**A**,**B**) Cartoon representation of the best scoring apo and holo state ensembles respectively from three independent simulations. Note that the holo state ensemble contains only two structures. Loop_427–446_ is shown in cyan, the catalytic serine is shown in red and the triglycine motif is shown in orange. Loop thickness corresponds to the relative weights of each structure in the ensemble. (**C**,**D**) Simulated distance distribution fits for the holo state (orange) overlaid with the experimental data (blue) for the apo (**C**) and holo (**D**) states. Simulated distributions are the weighted sum of simulations of the individual structures. The S137R1–S171R1 distance was omitted for the modeling of the holo state since it was restrained by the template crystal structures.

The ensembles in best agreement with experimental data are shown in Fig. 5 for both the *apo* and *holo* states. All three models of the *apo* state produced relatively heterogeneous loop structures. The remaining two ensemble models of the *apo* state are shown in Supplementary Fig. S9. Overall, the members of each *apo* state ensemble are slightly different except for one model, S004_01570, which was present in all three and notably was also the highest weighted member of all three *apo* ensembles. The lack of consistency in the members of each ensemble suggests that the modeling algorithm failed to converge for the *apo* state, which may reflect the inherently dynamic nature of the loop. Despite the apparently poor convergence, the ensemble models provide an envelope for the location of loop_427–446_ in the *apo* state and suggest a quasi-stable loop that occludes the active site. Cumulatively, these models are consistent with the broad distance distributions observed in the DEER experiments (Fig. 4) and the lack of electron density in both published crystal structures^25,26^. Given the experimental DEER data for the *lipo* state (Fig. 4, middle column), it appears likely that this distribution broadens ever further in the presence of a membrane substrate alone.

In contrast to the *apo* state, the *holo* state ensembles showed significantly better convergence. All three *holo* ensembles predominantly contained two structures (Fig. 4, right hand column), S004_2640 and S003_00341, with weights of approximately 0.6 and 0.4, respectively. One ensemble contained one additional structure at a weight of 0.01. Due to the low weight we did not consider this lone structure as significant and it is not discussed further. Unlike the *apo* ensembles, the *holo* ensembles are more structurally homogeneous. These models depict loop_427–446_ resting near the active site of ExoU. None of the models displayed the formation of a substrate binding pocket as observed in the VipD crystal structures, however this appears to be due to the presence of I170, which bridges the space between helix_168–176_ and loop_427–446._ As noted above, I170 is constrained to *apo* state structure coordinates despite the experimental observation of a significant conformational change at the adjacent site (S171R1) upon activation (Supplementary Fig. S6). I170 can easily be displaced by a relatively minor rotation or translation of helix_168–176_, which would then allow the formation of a deep pocket near the active site of ExoU. We also determined the simulated time domain traces of the ensemble models (Supplementary Fig. S10). These data compare reasonably well with the experimental traces, although slight deviations in the tail may indicate incomplete background correction for some traces.

## Discussion

Determining how proteins convert from inactive to active conformational states is essential for designing ligands that either enhance or interfere with these structural transitions. Here we use ExoU, a bacterial phospholipase synthesized by a particularly virulent subset of *P. aeruginosa* strains, as a model system to map regions of the molecule involved in activation of the enzyme. ExoU homologs and orthologs are encoded by several bacterial pathogens^42,43^, suggesting that knowledge of the activation mechanism could translate into inhibitory drugs against multiple Gram-negative pathogens. In support of this notion, experimental evidence is provided that VipD, synthesized by *Legionella pneumophila,* and ExoU share a conserved activation mechanism despite the use of distinctly separate eukaryotic cofactors, Rab5 and ubiquitin, respectively.

Unlike VipD, there is currently no evidence of a conformational change in ExoU upon interaction with ubiquitin alone. Instead, results to date indicate that ExoU activation requires synergistic events involving both substrate and cofactor^28^. Interestingly, VipD is not thought to exhibit interfacial activation^32^ which has previously been observed in PLA_2_ enzymes^34^ that are allosterically regulated by the lipid membrane^44^. While we have not explicitly tested ExoU for interfacial activation, it likely serves as a secondary regulation mechanism resulting in the cooperative activation of ExoU by both substrate and cofactor^28^. This hypothesis is supported by the observation that the addition of nanodiscs alone induced changes in ExoU structure near the catalytic site. This cooperative regulation is likely essential to prevent ExoU from degrading the *P. aeruginosa* membrane before it is injected into the host cell cytoplasm. Upon the addition of both ubiquitin and substrate relatively narrow distance distributions were observed that were significantly different from the *apo* state. These data suggest that ubiquitin allosterically stabilizes an active conformation of Loop_427–446_. Other notable differences between VipD and ExoU include the fact that helix_168–178_ of VipD is displaced compared to the ExoU crystal structure and there is no region of VipD analogous to residues 180–209 in ExoU. In all other PLA_2_ crystal structures that include coordinates for the catalytic aspartate, this location is occupied by two antiparallel β-strands orienting the catalytic aspartate towards the catalytic serine^31,32,34,45,46^. Residues of ExoU analogous to the β-structure preceding the catalytic aspartate are unresolved in the existing published crystal structures, but are predicted to form two β-strands^42^. While the investigation of this region is in progress, we hypothesize residues 180–209 must be displaced from their *apo* position to allow the ExoU catalytic aspartate and preceding residues to fold into the analogous β structure observed in other PLA_2_ enzymes.

Power saturation SDSL-EPR data of loop_427–446_ indicate that these residues fail to enter the membrane but still experience a change in solvent accessibility upon the addition of substrate. This observation further supports the hypothesis that the membrane serves as an allosteric activator for ExoU. It is important to consider the alternative explanation: this change in environment may be induced primarily by the localization to the membrane surface rather than allosteric conformational change. Notably these two possible explanations are not mutually exclusive, and the observed changes may be a combination of the two. The localization of these residues to the surface of the membrane implies a membrane orientation similar to what has been observed for other phospholipases^47–49^, and is consistent with previously proposed models of ExoU-membrane interaction^26,28^.

In addition to undergoing a conformational change upon formation of the *holo* state, our data show that loop_427–446_ is critical for ExoU activity. The most direct evidence testing our hypothesis was contributed by the cysteine disulfide bond formation experiment. The spontaneous formation of disulfide bonds by residues within the loop (T441C, N443C and T445C) and I170C indicates that these residues are relatively close in space, consistent with our computational models. The significant reduction in enzymatic activity for the disulfide-oxidized state suggests that movement of loop_427–446_ is required for activation. This finding is supported by the recovery of activity upon reduction with TCEP. The critical role of loop_427–446_ is further supported by the loss in activity that occurs upon the introduction of aspartate residues into the loop, consistent with the hypothesis that loop_427–446_ forms part of a hydrophobic pocket required for substrate interaction.

Our approach using SDSL-EPR and integrative modeling allowed us to obtain experimentally consistent models of the 20-residue ExoU loop_427–446_, which is a larger region than can currently be achieved with confidence by de novo modeling alone^39^. RosettaDEER allowed for the use of full DEER distance distributions to construct unresolved residues of the ExoU crystal structure, a relatively new capability that overcomes several obstacles when modeling proteins with DEER^40^. Ensemble modeling provided insights into the dynamic nature of ExoU loop_427–446_. Collectively, these methods provide a powerful approach for studying the dynamics of large, membrane associated systems which are often difficult to study using traditional structural techniques.

**Figure 6:**
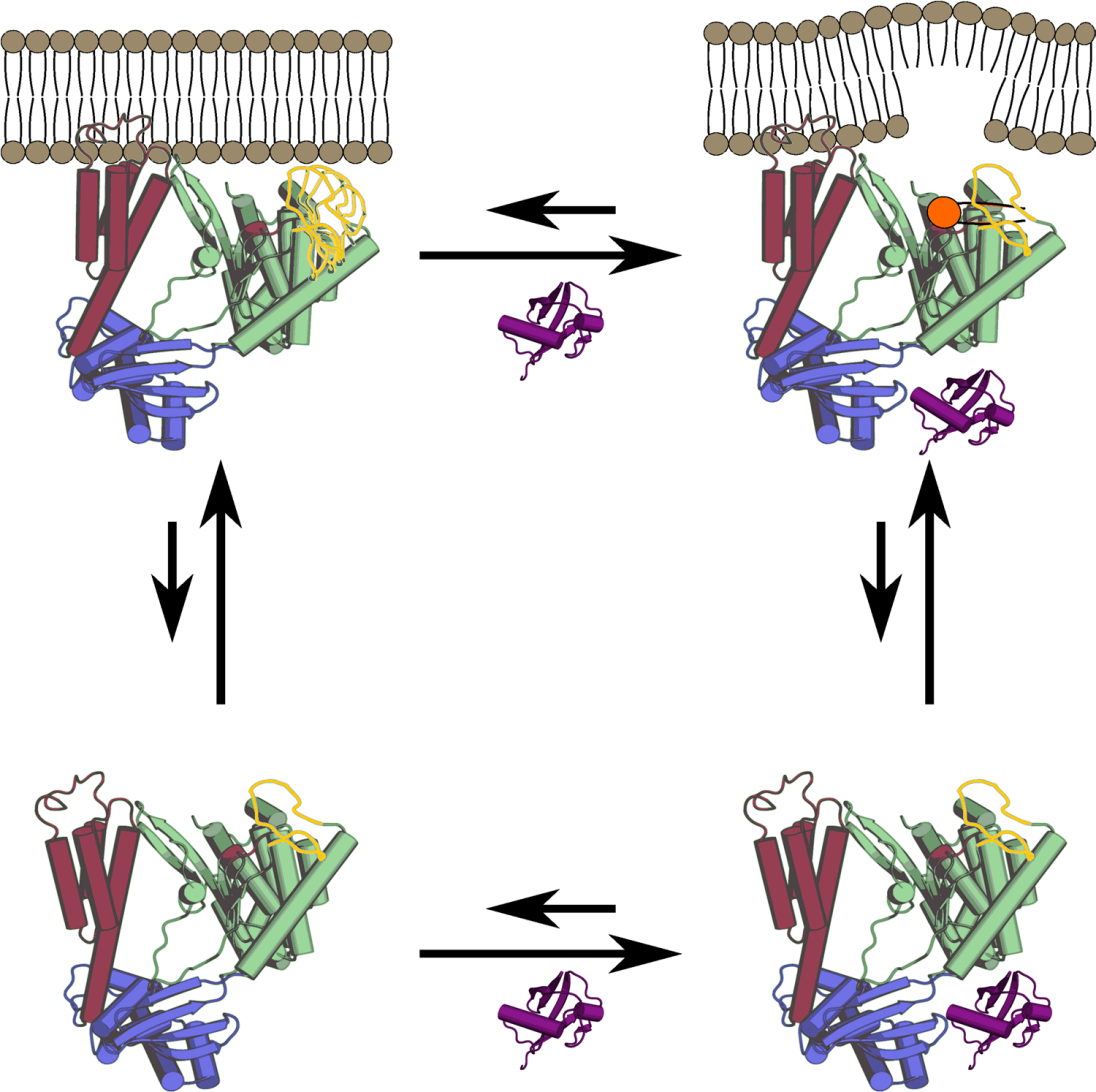
Activation model of ExoU. ExoU can associate with either the membrane or ubiquitin (purple) individually. Ubiquitin binds the ubiquitin binding domain (blue) but has no major effects in the absence of substrate. ExoU binds the membrane via its membrane localization domain (red)^28,29,59^ which places the catalytic domain (green, S142 shown as red sphere) near the membrane surface. Loop_427–446_ (yellow) unfolds and becomes disordered upon membrane association. Upon binding of ExoU to both the membrane and ubiquitin, loop_427–446_ adopts a new conformation that creates a substrate binding pocket (orange), resulting in hydrolysis and disruption of the membrane.

Cumulatively, our data imply an order of events for the activation of ExoU (Fig. 6). In this model, loop_427–446_ and helix_168–176_ cover the ExoU active site in the absence of substrate and cofactor. Although structural changes upon the addition of ubiquitin alone have yet to be observed, interaction of ExoU with a membrane substrate destabilizes the *apo* conformation of loop_427–446_ and possibly other regions of the membrane binding interface. Binding of ubiquitin to membrane-associated ExoU allosterically stabilizes loop_427–446_ in an active conformation that, along with helix_168–176_, forms a putative substrate binding pocket. Alternatively, membrane association of the ExoU-ubiquitin complex could also result in enzymatic activation, consistent with the synergistic effects of ubiquitin cofactor and substrate in the activation of ExoU.

## Materials and methods

### Mutagenesis and protein expression

All ExoU variants were constructed in pET15b with an N terminal 6X histidine tag and a thrombin cleavage site. Linear diubiquitin was constructed in the same vector with a native N-terminus. Point mutations were generated using the Q5 site directed mutagenesis kit (New England Biolabs) with primers designed using the NEBaseChanger application (https://nebasechanger.neb.com/). Reactions were transformed into chemically competent DH5α or NEBα per the manufacturer recommended protocol. Plasmids resulting from mutagenesis reactions were confirmed by double strand sequencing (Molecular Cloning Laboratories). All strains were cataloged in the Frank lab strain collection. Sequences are stored on the Frank lab shared data server. After validation, each construct was transformed into *Escherichia coli* BL21 (DE3) pLysS. Protein expression was induced with 0.25 mM IPTG in TERRIFIC broth after the cultures had reached mid-log phase in the same medium at 30˚ C. Cultures were harvested by centrifugation (12,000 × *g* at 4 °C, 10 min) and stored at – 80 °C until protein purification was initiated.

### Purification and spin labeling of ExoU

ExoU and ExoU-derivatives were purified essentially as previously described^28^. Bacterial pellets from cultures encoding recombinant, histidine-tagged ExoU were suspended in 20 ml lysis buffer (50 mM phosphate buffer, pH 7.3, 300 mM NaCl, 5 mM imidazole, 32 µg/ml benzamidine, 64 µg/ml leupeptin, 3.2 µg/ml pepstatin, and 32 µg/ml aprotinin). Cells were lysed by 3–5 passages through a French pressure cell (1000 psi) and clarified by centrifugation (30,000 × *g*, 4 °C, 20 min). Supernatants were applied to a gravity flow column with 3–5 ml TALON metal affinity resin (Takara) charged with cobalt and equilibrated with 30 ml binding buffer (50 mM phosphate buffer, pH 7.3, 300 mM NaCl, 5 mM imidazole). Bound protein was washed 3 × with 30 ml wash buffer (50 mM phosphate, pH 7.3, 300 mM NaCl, 5 mM imidazole) and eluted with 18 ml of elution buffer (50 mM phosphate, pH 7.0, 300 mM NaCl, 80 mM EDTA). Eluted protein was desalted into 50 mM phosphate, pH 7.0, 150 mM NaCl using two inline HiPrep 26/10 desalting columns (GE Life Sciences) on an AKTAexplorer 10 FPLC (GE Life Sciences). After desalting, the histidine tag was cleaved from each protein by the addition of 30 units of thrombin (MP Biochemicals #154163) from a 1 U/microliter stock solution in PBS and incubating at 4 °C for two hours while rocking gently. Thrombin was removed by passages over a 1 ml HiTrap benzamidine FF column (GE Life Sciences). Uncleaved protein and free histidine tag was purified away by reverse metal affinity chromatography of 3–5 ml of cobalt charged TALON metal affinity resin equilibrated with binding buffer. The flow through fraction was collected and glycerol was added to a final concentration of 20%. For spin labeling reactions *S*-(1-oxyl-2,2,5,5-tetramethylpyrroline-3-methyl) methanethiosulfonate (MTSL, Santa Cruz Biotechnology, Inc.) was added in fivefold excess of cysteine residues to be labeled, the protein preparation wrapped in foil and incubated overnight at 4 °C while rocking. Each preparation yielded between 10–40 mg spin labeled ExoU per liter of culture.

Purified protein was concentrated to approximately 1 ml by using Amicon Ultra-15 centrifugal concentrators (Millipore Sigma, 30 kDa cutoff). Size exclusion chromatography (SEC) was conducted using a Superdex 200 pg column [AKTA fast protein liquid chromatography (FPLC) system, GE Life Sciences] equilibrated with EPR buffer (20 mM Tris HCl, pH 7.5, 150 mM NaCl, 20% glycerol). Fractions containing ExoU variants were pooled and concentrated using the same filters. Proteins were diluted into 6 M guanidinium (pH 8.0) and concentrations were determined by absorbance at 280 nm using a SpectraMax M5 spectrometer (Molecular devices). Concentrations were calculated using extinction coefficients at 280 nm (ExoU 29.16 M^−1^ cm^−1^, diUb 2.56 M^−1^ cm^−1^) as calculated using the ExPASy ProtParam tool. Purified proteins were aliquoted, flash frozen in ethanol and dry ice and stored at – 80 °C until used.

### Purification of diubiquitin

Linear diubiquitin was purified as previously described with slight modifications^42^. Clones encoding linear diubiquitin were induced, suspended in 20 ml of 50 mM sodium acetate buffer (pH 7.3) with 32 µg/ml benzamidine, 64 µg/ml leupeptin, 3.2 µg/ml pepstatin, and 32 µg/ml aprotinin and lysed in a French pressure cell (3–5 passes). Cell lysates were clarified by centrifugation (30,000 × *g*, 4 °C, 20 min). The pH was adjusted to ~ 4.5 by stepwise addition of glacial acetic acid and mixed with an additional 20 ml of 50 mM sodium acetate buffer (pH 4.5). Precipitated debris was removed by centrifugation (30,000 × *g*, 4 °C, 20 min) and the supernatant was passed through a 0.2-micron filter. Filtered supernatants were subjected to cation exchange chromatography using an HiPrep SP FF 16/10 column (GE Life Sciences) on the AKTA FPLC system. Ubiquitin was eluted using a sodium chloride gradient (0–500 mM). Fractions were analyzed via SDS-PAGE and Coomassie staining. Fractions containing ubiquitin were pooled and concentrated to 2–5 ml using an Amicon Ultra-15 centrifugal concentrator (Millipore Sigma) with a 10 kDa cutoff. Concentrated ubiquitin was purified via size exclusion chromatography on a sephacryl S-200 column equilibrated with EPR buffer. Fractions containing ubiquitin were pooled, concentrated and quantified by absorbance at 280 nm as described above. Ubiquitin was aliquoted stored at – 80 °C until needed.

### Preparation of nanodiscs and large unilamellar vesicles

All phospholipids were purchased from Avanti Polar Lipids. Nanodisc were prepared with 5.44 μmol POPC (~ 78 mol %), 1.36 μmol POPG (20 mol %), 0.136 μmol PI(4,5)P_2_ (2 mol %) and 0.017 μmol Rhodamine PE (~ 0.25 mol %). Phospholipids were dried under nitrogen and then subjected to a vacuum overnight. Dried phospholipids were suspended in 200 μl of buffer (20 mM Tris HCl, pH 7.5, 120 mM sodium cholate, 0.5 mM EDTA, 100 mM NaCl) and sonicated until clear. After sonication, phospholipids were chilled on ice for 5 min. After cooling 2.5 mg (~ 100 nmol) MSP1D1 (Sigma-Aldrich) was added resulting in a final ratio of approximately 65 lipids per MDP1D1 monomer^50^. The mixture was incubated at room temperature while gently rocking for 1 h. The mixture was dialyzed (slide-a lyzer cassette, 0.5–3.0 ml, 10 kDa cutoff, Thermo Fisher Scientific) against 2–4 l of dialysis buffer (20 mM Tris, pH 7.5, 150 mM NaCl, 0.5 mM EDTA) overnight. The following day, dialysis was continued with at least three additional changes of the dialysis buffer. The resulting nanodiscs were concentrated using an Microcon Ultracel YM-30 (Millipore) centrifugal concentrator. Characterization by dynamic light scattering (Zetasizer, Malvern Instruments) indicated a homogeneous population with a diameter slightly greater than 10 nm (Supplementary Fig. S11), as expected for MSP1D1 nanodiscs^50^. The final lipid concentration was determined based on the absorbance of Rhodamine PE at 573 nm and an extinction coefficient of 88 mM^−1^ cm^−1^. Nanodiscs were stored at 4 °C and used within 2 weeks of preparation.

Liposomes (large unilamellar vesicles) with a lipid composition identical to the nanodiscs were prepared by extrusion through 0.1 micron filters as previously described^28^.

### Double electron–electron resonance

DEER samples in 1.1 × 1.6 mm glass capillaries (VitroCom) contained approximately 100 μM spin labeled ExoU in a final volume of 16 μl. *Lipo* and *holo* samples contained nanodisc bilayers at a final concentration of 20 mM lipid. *Holo* and diUb samples contained linear diubiquitin at ≥ 600 μM. All samples contained 25% (vol/vol) of perdeuterated glycerol (Sigma-Aldrich) as cryoprotectant. Samples were flash frozen in a dry ice-acetone bath and immediately inserted into the sample resonator.

All DEER experiments were conducted at Q band (∼ 33 GHz) using an ELEXSYS E580 spectrometer (Bruker BioSpin) equipped with an EN 5107D2 resonator and 10-W microwave amplifier. The resonator was kept at – 80 °C using an Oxford cryostat. Four-pulse DEER patterns^7^ were applied using 32 ns pump pulses at the low field maximum of the Q band spectrum and 16 ns π/2 observer pulses 54 MHz upfield. Signals were averaged from 4–24 h. Analysis of DEER data was conducted using DeerAnalysis 2018^51^. All spectra were background corrected using a homogeneous 3D background model and fit using Tikhonov regularization. All smoothing parameters were chosen using generalized cross validation. Error bands were calculated using the default validation protocol implemented in DeerAnalysis 2018. The resulting data were plotted using Python 3.6 and Matplotlib.

### ExoU activity assays

Activity assays were conducted as previously described^42^. Briefly, 50 μM N-((6-(2,4-dinitrophenyl)amino)hexanoyl)-2-(4,4-difluoro-5,7-dimethyl-4-bora-3a,4a-diaza-*s*-indacene-3-pentanoyl)-1-hexadecanoyl-*sn*-glycero-3-phosphoethanolamine (PED6) was used except for PED6 titrations and 20 μM diubiquitin was used as cofactor. PED6 titrations were conducted using seven twofold dilutions starting at 200 μM. Reactions were initiated by adding ExoU to a final concentration of 10 nM. Fluorescent readings were taken every minute for 1 h beginning immediately after initiation. Fluorescent signal was normalized to 0.5 nmol BODIPY or to WT ExoU for comparison. For disulfide cross linked ExoU, proteins were preincubated with 2 mM TCEP for 30 min when applicable. All activity assays were conducted in triplicate. Analysis of activity data was conducted using Python 3.6. Linear ranges were fit, and the slope used as the catalytic rate. For PED6 titrations, slopes were fit to the Michalis-Menten equation to determine catalytic constants *K*_*m*_ and *V*_*max*_.

### Continuous wave EPR

Continuous wave EPR experiments were performed at X-band on an Elexsys E500 spectrometer (Bruker Biospin, Billerica, MA) as previously described^28^ with the following modifications: 50 μM spin labeled ExoU in 20 mM Tris, 150 mM NaCl, 20% glycerol (vol/vol) at pH 7.5 was analyzed alone or with the addition of with 500 μM diUb, 10 mM nanodiscs, or both. Field sweeps were performed with the following spectrometer settings: 10 mW microwave power, 5.12 ms time constant, 20.97 s scan time, 20.48 s conversion time, 100-kHz field modulation, 1.0 Gauss field modulation amplitude, 100 Gauss sweep width as previously described^28^. Field sweeps were averaged over 15 scans. All CW traces were normalized to the area under the curve. All plots and analysis were performed using Python3.6 and Matplotlib.

### Power saturation EPR

Power saturation experiments were conducted as previously described^28^. All samples contained spin labeled ExoU at 100–150 μM. *Lipo* and *holo* samples contained a final concentration of 10 mM lipid (~ 0.5 mM nanodiscs), and holo samples contained 1 mM diubiquitin. Data were collected under the flow of compressed air (20% O_2_), N_2_ gas or N_2_ gas in the presence of 5–10 mM NiEDDA. Accessibility parameters, Π(O_2_) and Π(NiEDDA), were calculated as previously described^52^, and Π(NiEDDA) values were normalized to 20 mM NiEDDA^53^. The EPR depth parameter (Φ) was calculated as previously described^38,52,53^.

### Protein and ensemble modeling

The protein and ensemble modeling process is illustrated in Fig. S8. Full length ExoU was modelled using Rosetta 3.10^54^ with the RosettaDEER^40^ module. The Rosetta hybridize mover^41^ was used to model approximately 10,000 ExoU structures using the ExoU and *pf*ExoU crystal structures (PDB: 3TU3, 4QMK) as templates supplemented with DEER distance distributions as restraints (see Supplementary procedure 1) and fragment libraries generated by the Robetta web server^55^. The top 500 scoring models (by Rosetta score and RosettaDEER distance distribution satisfaction) were then used as a pool to build an ensemble model.

The top 500 scoring models obtained using the above methods were used as a sampling pool to fit an ensemble model. For ensemble modeling, distance distributions were simulated for every structure in the model pool. All simulated distance distributions for ensemble modeling and Fig. S7 were performed using a python implementation of the rotamer library approach used in MMM^56,57^ with the R1A_298K rotamer library from MMM 2018. Briefly, the rotamer library is superimposed on the backbone of each site. For each rotamer, non-bonded interactions of all side chain atoms with all other atoms within 15 Å of the residue Cα carbon are calculated using a softened Lennard–Jones potential^57^. Rotamers are then reweighted by the Boltzmann distribution:

$$ w_{new} = w_{old} \times e^{{ - E_{lj} /kT}} , $$

where *w*_*new*_ is the new weight, *w*_*old*_ is the R1A_298K rotamer library^56^ weight, *E*_*lj*_ is the sum of the Lennard–Jones potentials (J) of all atoms of the rotamer, *k* is the Boltzmann constant in J/(mol K) and T is the temperature (298 K). After reweighting, the rotamer libraries are pruned to remove insignificant rotamers and normalized to sum to 1. Pairwise spin–spin distances, ***d***, and distance weights, ***w***, between the remaining rotamers of each site are calculated

$$ d_{ij} = \sqrt {\left( {x_{i} - x_{j} } \right)^{2} + \left( {y_{i} - y_{j} } \right)^{2} + \left( {z_{i} - z_{j} } \right)^{2} } , $$

$$ {\varvec{w}}_{ij} = w_{i} \times w_{j} . $$

For all rotamers *i* of site 1 and *j* of site two, where *x*_*i*_*, y*_*i*_ and *z*_*i*_ are the cartesian coordinates of the spin center or rotamer *i* and *w*_*i*_ the is the relative population of rotamer *i.* A weighted histogram is then made using ***d*** and ***w,*** which is convolved with a normal distribution with a variance of 1 Å. The resulting distance distribiton, *P(r)*, is normalized such that

$$ \smallint P\left( r \right)dr = 1. $$

Time domain signals of simulated distance distributions were calculated using modulation depth and background decay constants fit with DEERAnalysis 2018.

While other unresolved loops of ExoU were modeled by Rosetta for completeness, they were not considered when simulating distance distributions for ensemble modeling due to high variability and low confidence in their modeled structures. These simulations were then used to fit to the experimental data using a Monte Carlo sampling approach. For 500,000 iterations 10 structures were randomly selected from the pool of 500 and assigned uniform weights. The ensemble was then scored against the experimental data using the Jaccard index^58^. The Jaccard index scores the similarity of two sets, sets, **p** and **q**, between 0 and 1 with 1 being perfect overlap and 0 being no overlap. The Jaccard index is defined as

$$ J\left( {{\varvec{p}},{\varvec{q}}} \right) = \frac{{ \mathop \sum \nolimits_{i} min\left( {p_{i} ,q_{i} } \right)}}{{\mathop \sum \nolimits_{i} max\left( {p_{i} ,q_{i} } \right)}} . $$

A subroutine was then used to optimize weights. For each iteration of the subroutine, new weights were randomly sampled from the Dirichlet distribution initialized from the previously optimal weights. If the new weights resulted in an improved fit to the experimental DEER data, they were stored as the optimal weights otherwise they were discarded. In the event that any structure fell below a weight of 0.001 it was discarded from the ensemble to drive the algorithm towards a minimal set of structures. This subroutine was repeated until there were no improvements made for 100 iterations. The whole Monte Carlo algorithm was run three times with both the *apo* and *holo* constraints to assess convergence.

## Supplementary information

Supplementary Information.
