# Supplementary material for: Characterization of the ExoU activation mechanism using EPR and integrative modeling
Source: Sci Rep. 2020 Nov 12;10:19700. doi: 10.1038/s41598-020-76023-3 (PMC7665212; doi:10.1038/s41598-020-76023-3)
Supplement: Supplementary file 1 — Supplementary Information. [file 41598_2020_76023_MOESM1_ESM.docx]

Characterization of the ExoU activation mechanism using EPR and integrative modeling

Maxx H. Tessmer^1^, Samuel A. DeCero^2^, Diego del Alamo^3,4^, Molly O. Riegert^2^, Jens Meiler^3,5^, Dara W. Frank^2*^ and Jimmy B. Feix^6^

^1^ Department of Chemistry, University of Washington, Seattle WA, USA

^2^ Department of Microbiology & Immunology, Medical College of Wisconsin, Milwaukee WI, USA

^3^Department of Chemistry and Center for Structural Biology, Vanderbilt University, Nashville TN, USA

^4^Department of Molecular Physiology and Biophysics, Vanderbilt University, Nashville TN, USA

^5^Institute for Drug Discovery, Leipzig University Medical School, Leipzig SAC, Germany

^6^Department of Biophysics, Medical College of Wisconsin, Milwaukee WI, USA

*Corresponding Author

Corresponding Author's email address: frankd@mcw.edu

SUPPLEMENTAL INFORMATION

**SUPPLEMENTAL INFORMATION**


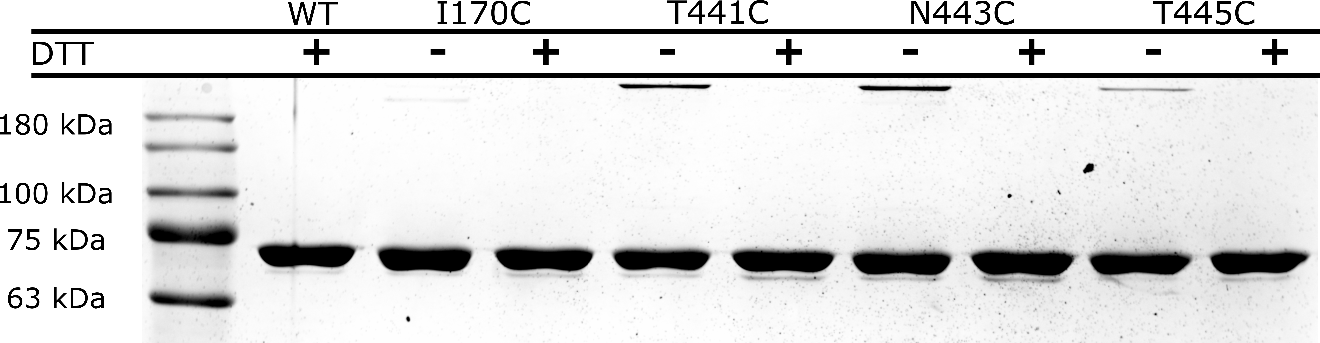


**Figure S1. EMSA of ExoU single cysteine mutant controls.** EMSA of ExoU disulfide cross links between single cysteine mutants in the presence and absence of DTT. The image was cropped as stained bands were not detected below the 63 kDa marker.


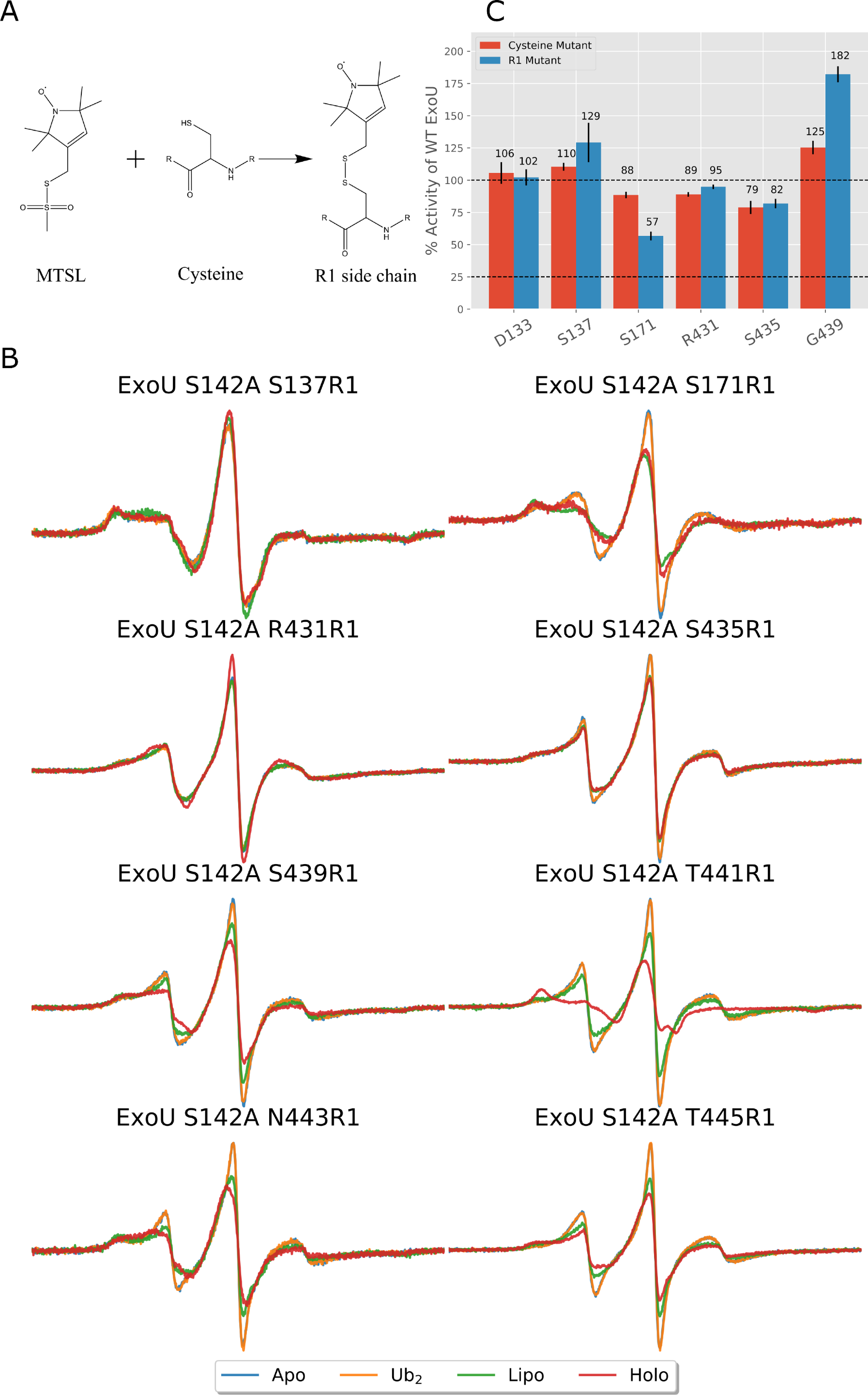


**Figure S2.**  **CW EPR of spin labeled single cysteine ExoU loop_427-446_ variants.** A) MTSL Spin labeling reaction. B) CW spectra were taken in the apo state (blue), in the presence of 500 µM diubiquitin (orange), 10 mM nanodiscs (green) or both diUb and nanodiscs (holo state, red). C) Enzymatic activities of cysteine point mutants and their spin labeled derivatives relative to wild type ExoU. Note that activities of the remaining cysteine mutants are shown in Figure 2B.


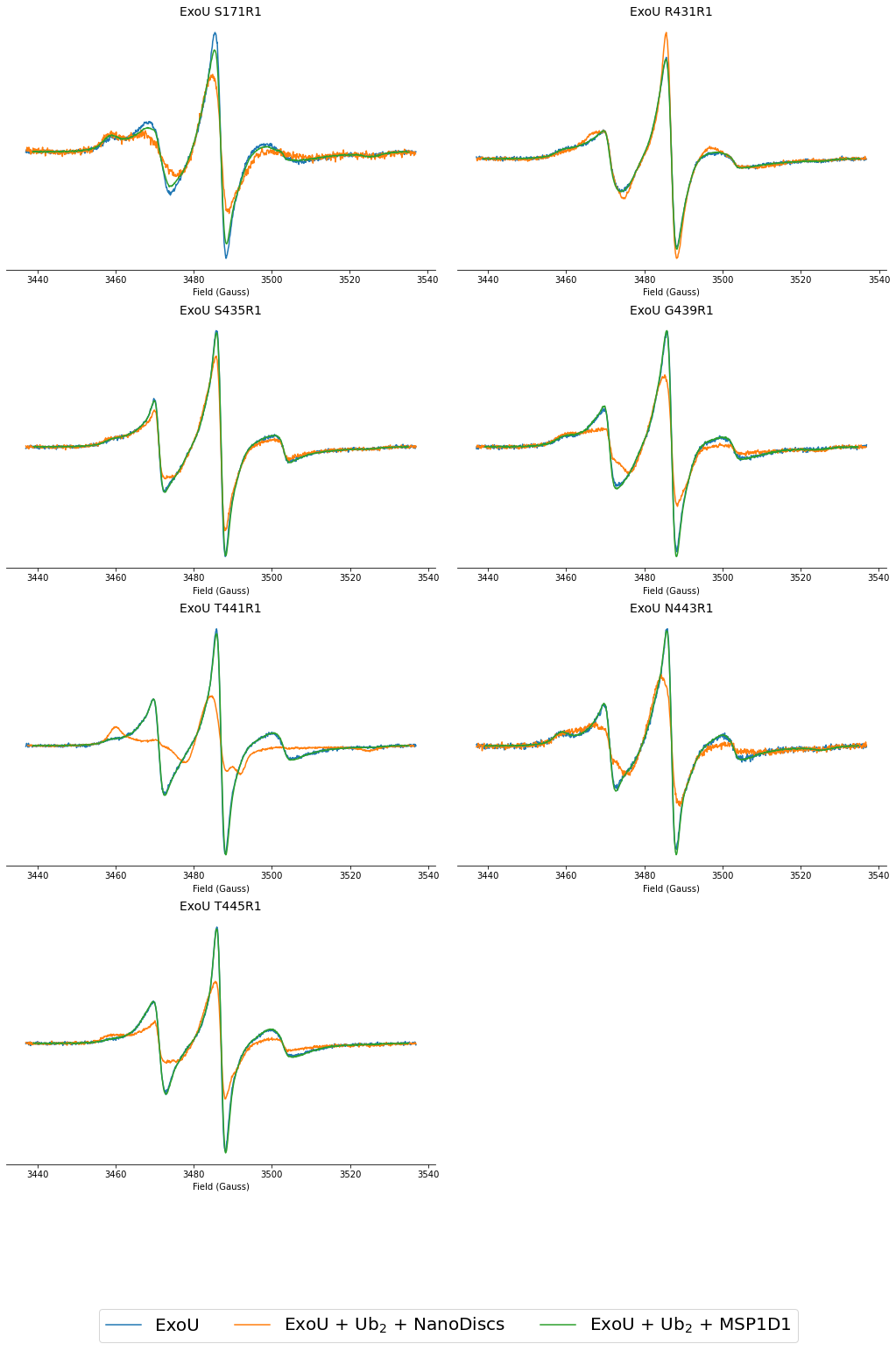


**Figure S3.** **MSP1D1 in solution does not affect ExoU loop_427-446_ variants**. CW EPR spectra of spin labeled ExoU loop_427-446_ variants in the presence of MSP1D1 in solution (green trace) are unchanged relative to ExoU in the *apo* state (blue trace). Significant changes observed in the *holo* state (orange trace)are shown for comparison.


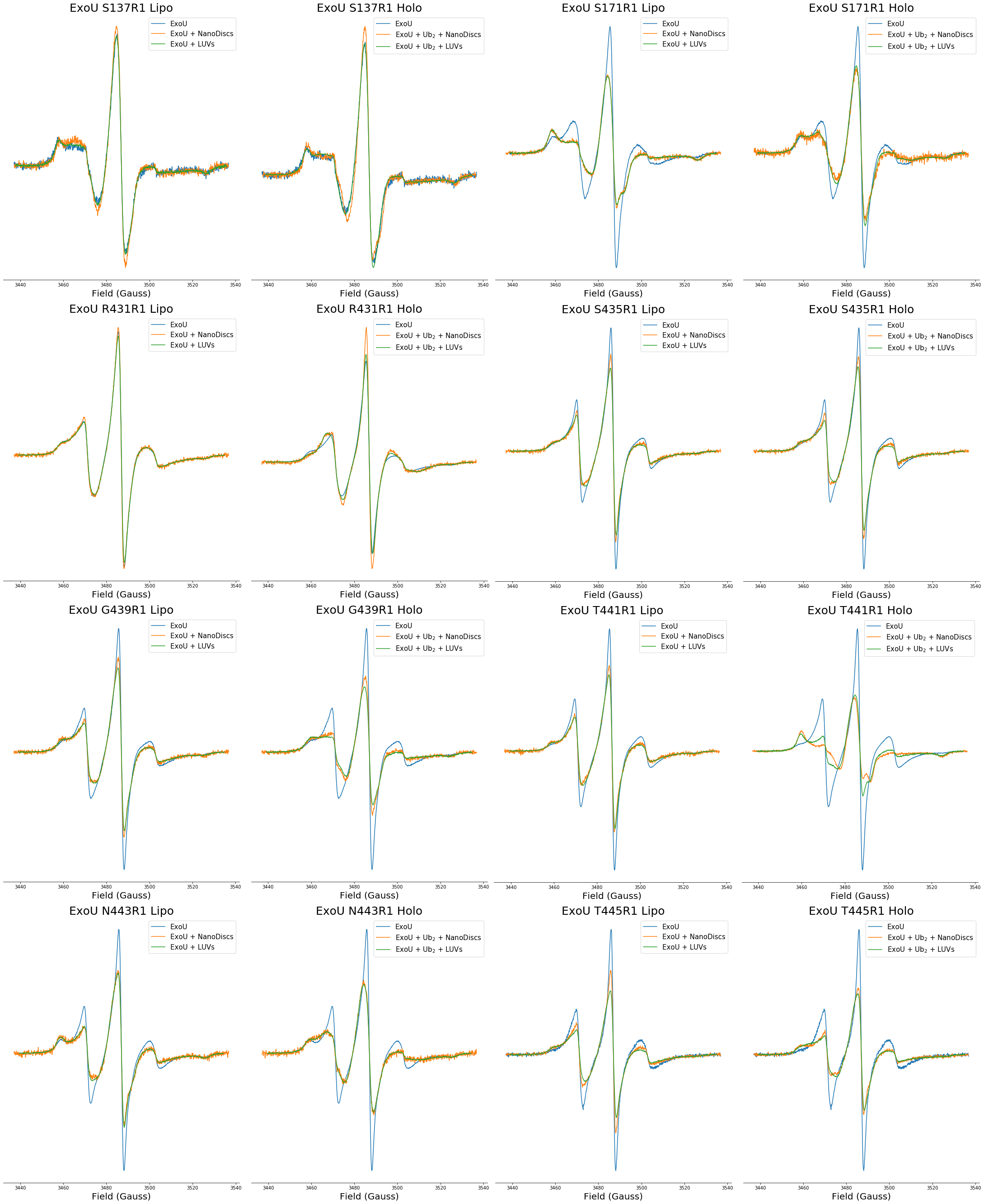


**Figure S4**. **CW EPR spectra of spin labeled ExoU loop_427-446_ variants in nanodiscs and LUVs.** EPR spectra of the *lipo* and *holo* states prepared with nanodiscs (orange traces) are compared to those prepared with LUVs (green traces). *Apo* state EPR spectra (blue traces) are shown for comparison. With the exception of *holo* state T441R1, EPR spectra obtained with nanodiscs and LUVs are essentially identical.


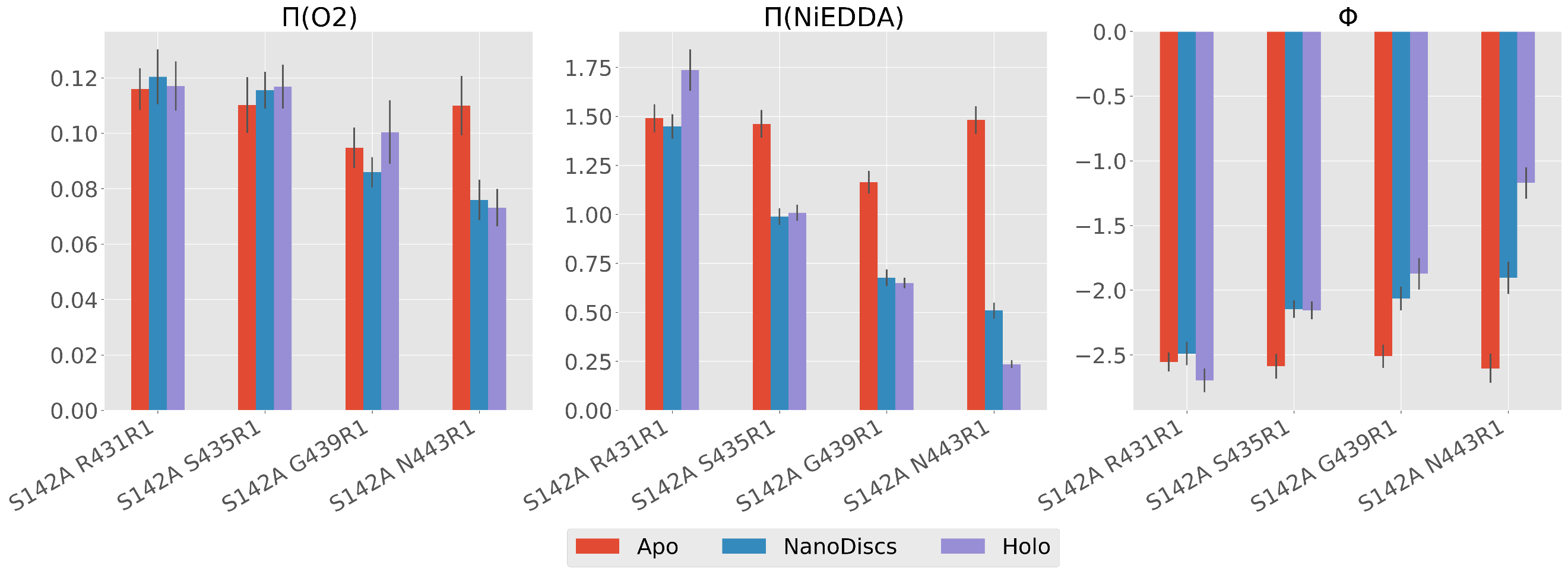


**Figure S5. Power saturation EPR data for spin labeled single cysteine ExoU loop_427-446_ variants.** Oxygen and NiEDDA accessibility parameters Π(O_2_) and Π(NiEDDA), respectively, and Φ values for four sites of ExoU loop_427-446_. Error bars indicate standard deviations calculated using bootstrap statistics.


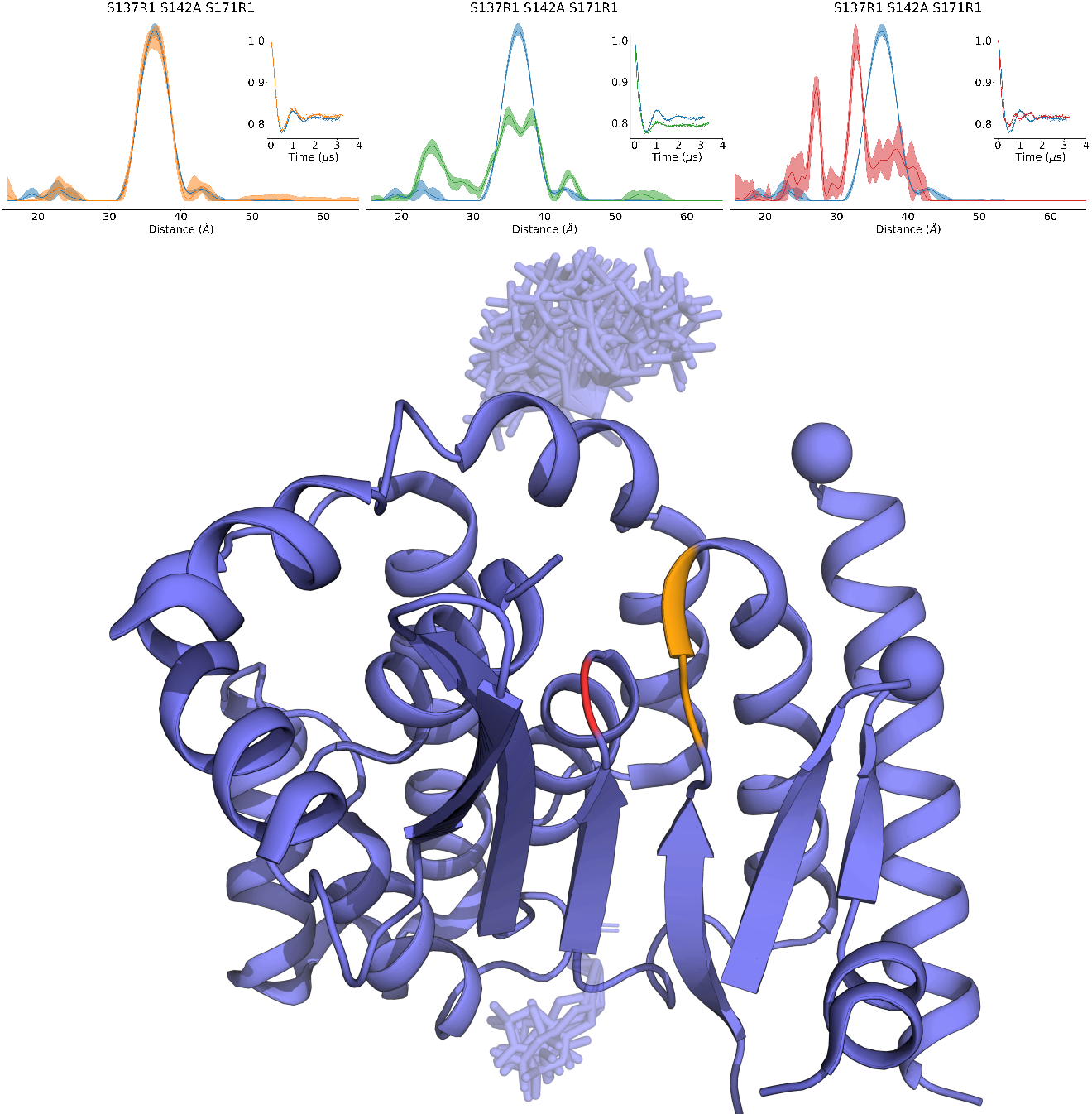


**Figure S6.** **DEER distance distributions for ExoU 137R1-S142A-S171R1**. DEER distance distributions were determined for double spin labeled ExoU 137R1-S142A-S171R1 in the apo state (blue), in the presence of diubiquitin alone (orange), in the presence of nanodiscs alone (green) or in the presence of both (red).


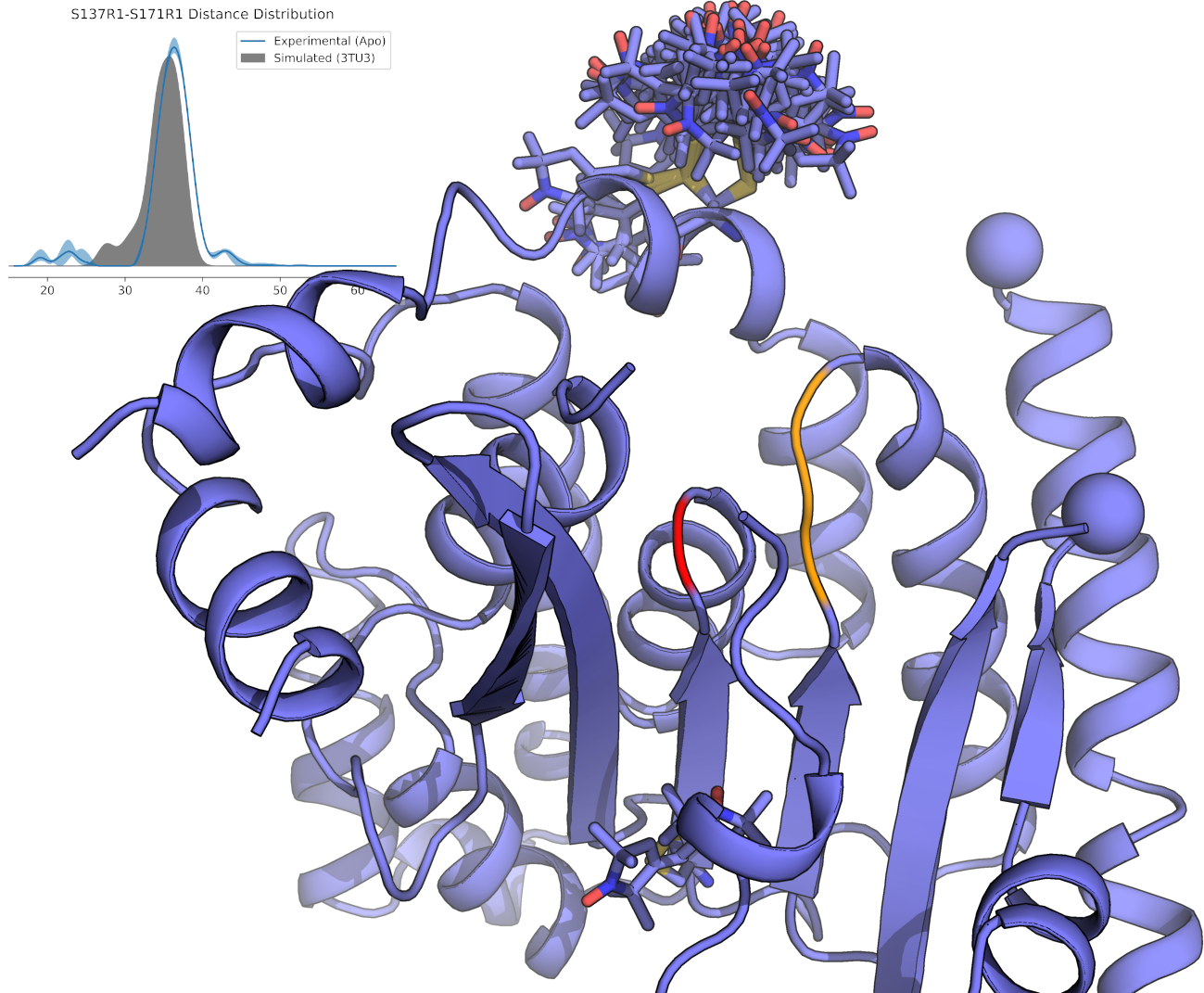


**Figure S7.** Locations and distributions of the simulated spin label side chain at S171R1 and S137R1 in the apo state and the predicted and experimental distance distributions (inset). The catalytic serine is shown in red and the triglycine motif is orange.


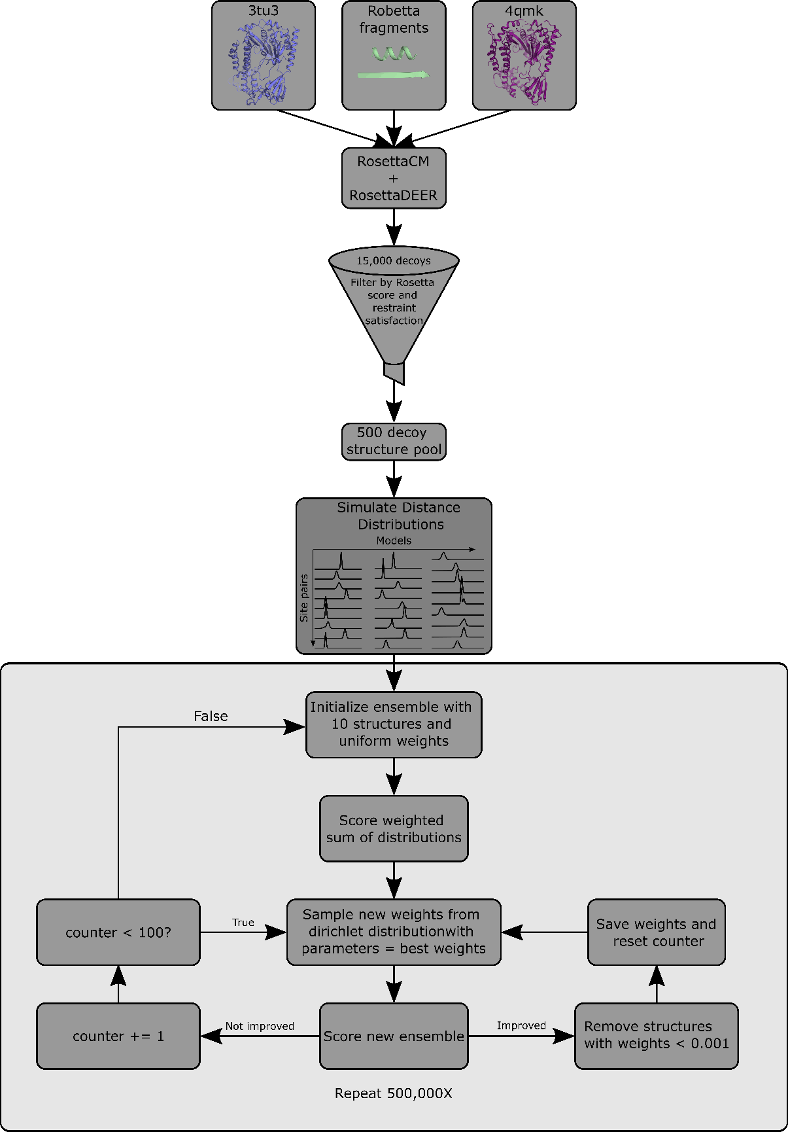


**Figure S8.** **Protein ensemble modeling flow chart.**  Initial Rosetta modeling with RosettaCM and RosettaDEER employed DEER distance distributions from spin label pairs as previously described ^1^. Ensemble optimization employed simulated distance distributions for the decoy pool and DEER data for loop_427-446_ from the present work.


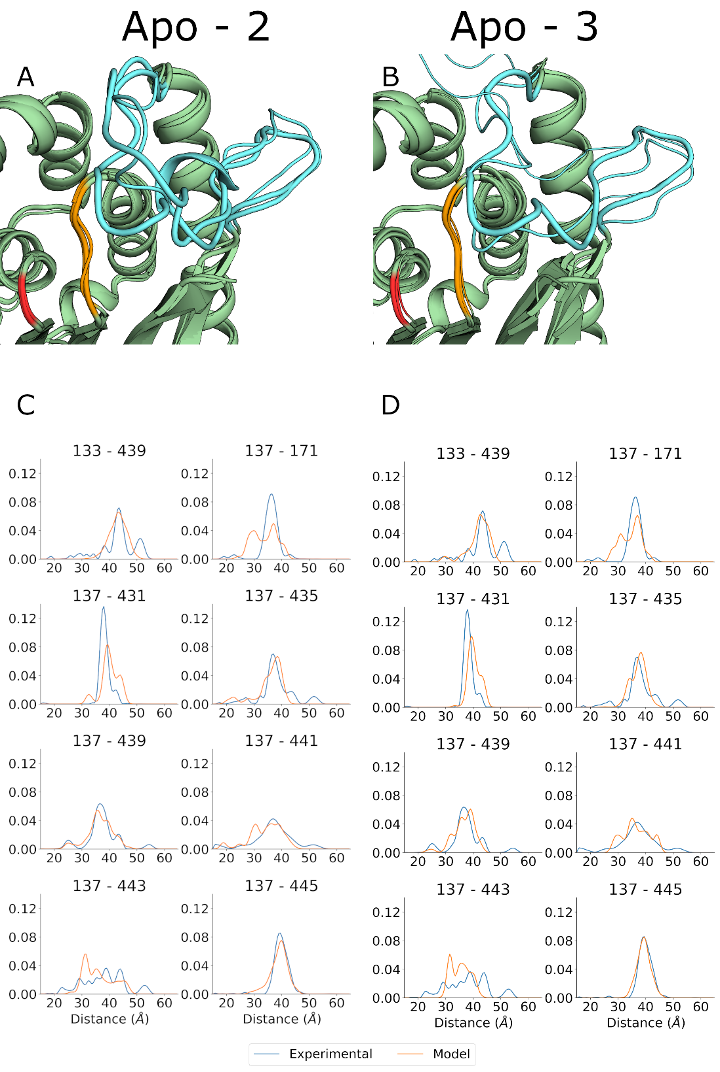


**Figure S9.**  **ExoU loop_427-446_ ensemble modeling replicates**. Replicates of ensemble modeling results. A-B) Cartoon representation of the remaining two Apo ensemble modes. Loop_427-446_.is shown in cyan, the catalytic serine is shown in red and the triglycine motif is shown in orange. Loop thickness corresponds to the relative weights of each structure in the ensemble. C-D) Simulated distance distribution fits for the holo state (orange) overlaid with the experimental data (blue). All holo ensemble models were effectively the same as the model shown in Fig 4.


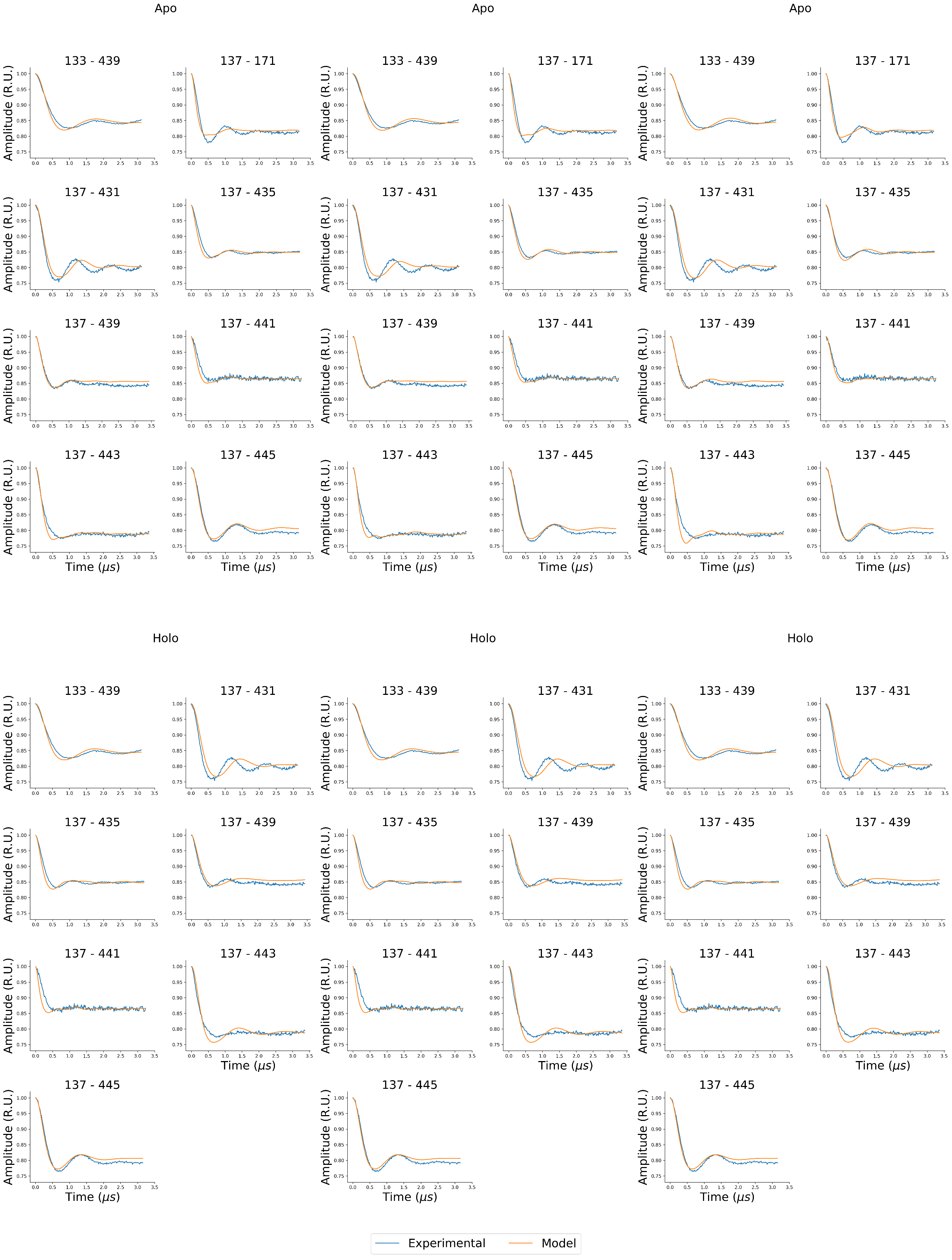
**Figure S10.** Comparison of experimental (blue) and calculated (orange) DEER dipolar evolution data.


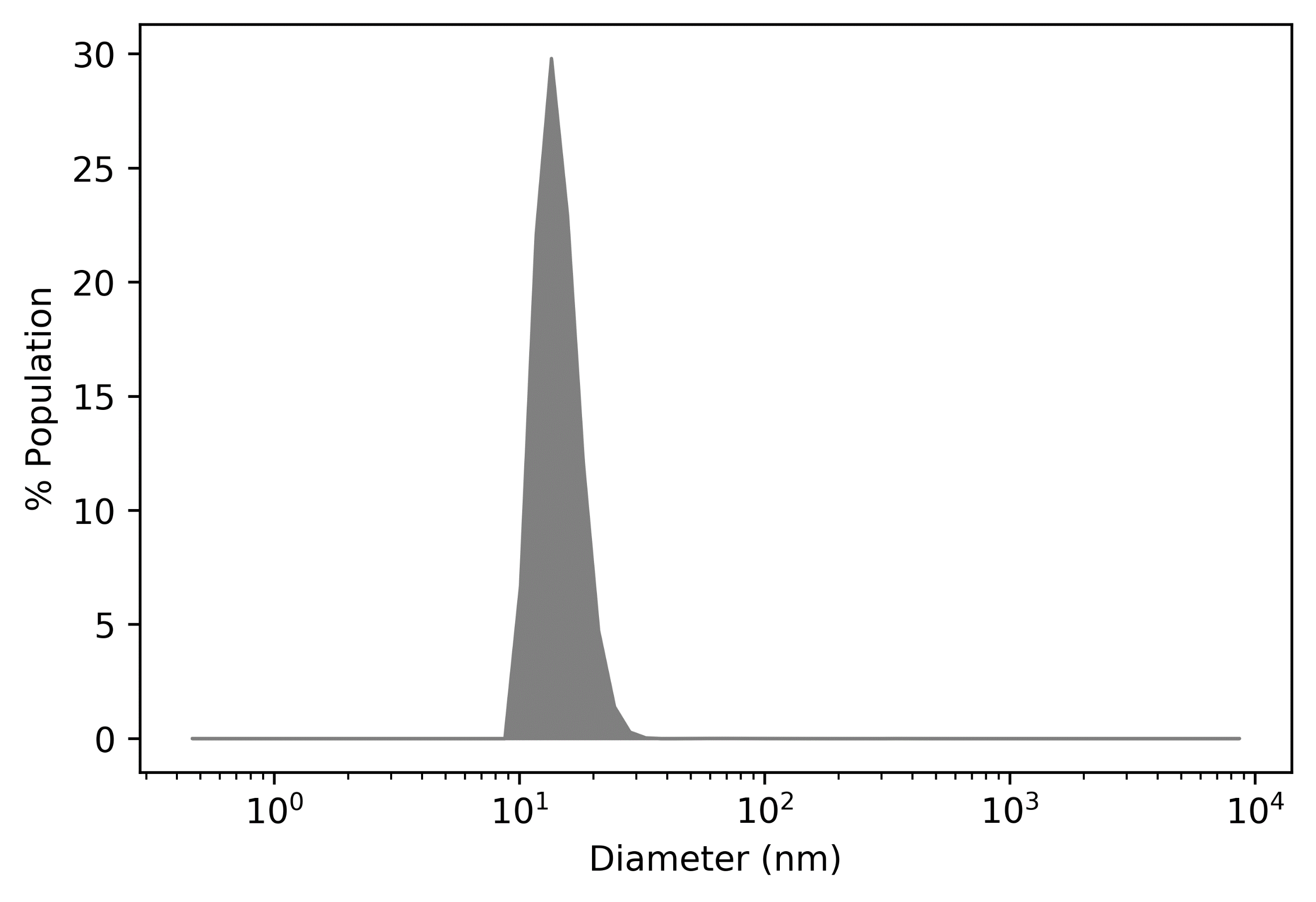
**Figure S11.** Dynamic light scattering of nanodiscs**.**

1. Del Alamo, D. et al. Rapid simulation of unprocessed DEER decay data for protein fold prediction. *Biophysical Journal* (2019).
